# KEYLINK: towards a more integrative soil representation for inclusion in ecosystem scale models. I. review and model concept

**DOI:** 10.7717/peerj.9750

**Published:** 2020-09-09

**Authors:** Gaby Deckmyn, Omar Flores, Mathias Mayer, Xavier Domene, Andrea Schnepf, Katrin Kuka, Kris Van Looy, Daniel P. Rasse, Maria J.I. Briones, Sébastien Barot, Matty Berg, Elena Vanguelova, Ivika Ostonen, Harry Vereecken, Laura M. Suz, Beat Frey, Aline Frossard, Alexei Tiunov, Jan Frouz, Tine Grebenc, Maarja Öpik, Mathieu Javaux, Alexei Uvarov, Olga Vindušková, Paul Henning Krogh, Oskar Franklin, Juan Jiménez, Jorge Curiel Yuste

**Affiliations:** 1 Department of Biology, Plants and Ecosystems (PLECO), Universiteit Antwerpen, Antwerpen, Belgium; 2 Biogeography and Global Change, National Museum of Natural Sciences-Spanish National Research Council (MNCN-CSIC), Madrid, Spain; 3 Institute of Forest Ecology, Department of Forest and Soil Sciences, University of Natural Resources and Life Sciences (BOKU), Vienna, Austria; 4 Biogeochemistry Group, Forest Soils and Biogeochemistry, Swiss Federal Research Institute WSL, Birmensdorf, Switzerland; 5 CREAF, Cerdanyola del Vallès, Spain; 6 Universitat Autònoma de Barcelona, Cerdanyola del Vallès, Spain; 7 Agrosphere Institute, IBG, Forschungszentrum Jülich GmbH, Jülich, Germany; 8 Institute for Crop and Soil Science, Julius Kühn-Institut (JKI), Braunschwei, Germany; 9 OVAM, Flemish Institute for Materials and Soils, Mechelen, Belgium; 10 Department of Biogeochemistry and Soil Quality, Norwegian Institute of Bioeconomy Research (NIBIO), Aas, Norway; 11 Departamento de Ecología y Biología Animal, Universidad de Vigo, Vigo, Spain; 12 Institute of Ecology and Environmental Sciences, IRD, UPEC, CNRS, INRA, Sorbonne Université, Paris, France; 13 Department of Ecological Science, Vrije Universiteit Amsterdam, Amsterdam, Netherlands; 14 Groningen Institute for Evolutionary Life Sciences, University of Groningen, Groningen, Netherlands; 15 Forest Research, Alice Holt Lodge, Farnham, UK; 16 Institute of Ecology and Earth Sciences, University of Tartu, Tartu, Estonia; 17 Comparative Plant and Fungal Biology, Royal Botanic Gardens, Kew, London, UK; 18 Forest Soils and Biogeochemistry, Swiss Federal Research Institute WSL, Birmensdorf, Switzerland; 19 A.N. Severtsov Institute of Ecology and Evolution RAS, Moscow, Russia; 20 Institute for Environmental Studies, Charles University, Prague, Czech Republic; 21 Slovenian Forestry Institute, Ljubljana, Slovenia; 22 Earth and Life Institute, UCLouvain, Louvain-la-Neuve, Belgium; 23 Department of Bioscience, Aarhus University, Silkeborg, Denmark; 24 Department of Forest Ecology and Management, Swedish University of Agricultural Sciences, Umeå, Sweden; 25 International Institute for Applied Systems Analysis IIASA, Laxenburg, Austria; 26 Department of Biodiversity Conservation and Ecosystem Restoration, ARAID/IPE-CSIC, Jaca, Spain; 27 BC3-Basque Centre for Climate Change, Scientific Campus of the University of the Basque Country, Bilbao, Bizkaia, Spain; 28 IKERBASQUE, Basque Foundation for Science, Bilbao, Spain

**Keywords:** Soil fauna, Model, Soil organic matter (SOM), Hydrology, Pore size distribution (PSD), Soil biota, Ecosystem

## Abstract

The relatively poor simulation of the below-ground processes is a severe drawback for many ecosystem models, especially when predicting responses to climate change and management. For a meaningful estimation of ecosystem production and the cycling of water, energy, nutrients and carbon, the integration of soil processes and the exchanges at the surface is crucial. It is increasingly recognized that soil biota play an important role in soil organic carbon and nutrient cycling, shaping soil structure and hydrological properties through their activity, and in water and nutrient uptake by plants through mycorrhizal processes. In this article, we review the main soil biological actors (microbiota, fauna and roots) and their effects on soil functioning. We review to what extent they have been included in soil models and propose which of them could be included in ecosystem models. We show that the model representation of the soil food web, the impact of soil ecosystem engineers on soil structure and the related effects on hydrology and soil organic matter (SOM) stabilization are key issues in improving ecosystem-scale soil representation in models. Finally, we describe a new core model concept (KEYLINK) that integrates insights from SOM models, structural models and food web models to simulate the living soil at an ecosystem scale.

## Introduction

Soils are multi-scale complex systems that contribute to several ecosystem services such as food, fibre and fuel production, carbon sequestration or water regulation (Adhikari & Hartemink, 2016). Soil quality has many definitions (see review by Bünemann et al. (2018)) which however all agree on the interconnected importance of soil organic matter (SOM) and soil structure for soil functioning. Soil quality can decline rapidly in response to disturbance and management changes such as industrial and agricultural activities, deforestation, overgrazing, pollution, and overexploitation for fuelwood (Oldeman, Hakkeling & Sombroek, 1991, see also review by Gregory et al. (2015)). Soil can sometimes recover fast (Hirsch et al., 2017), but mismanagement can limit its regeneration and buffering capacity resulting in limited recovery after soil degradation (see review by Gomiero (2016)). Decline of SOM threatens soil functioning in many ways affecting soil fertility, productivity, and food security, as well as the stabilization or reduction of atmospheric CO_2_ levels (Gobin et al., 2011).

The full impact of a range of management or environmental changes on soil and ecosystem functioning can only be predicted with mechanistic models in which key mechanisms are sufficiently represented (Vereecken et al., 2016). Mechanistic models allow us to integrate our knowledge of the soil system gained from numerous experiments and also to test its current level as reflected by the models’ predictive capability.

For stand/ecosystem predictions, a very limited number of soil models are usually used, mainly based on CENTURY (Parton et al., 1987; Paustian, Parton & Persson, 1992), ROTHC (Jenkinson & Rayner, 1977), and few others (Liski et al., 2005; review: Campbell & Paustian, 2015) although a wide range of other models have been developed (Manzoni & Porporato, 2009; Reichstein et al., 2003; Sándor et al., 2017). Essentially, most models describe the soil as consisting of homogeneous horizons, where SOM transformation occurs in a cascade from easily degradable to passive or stable SOM based on its chemical complexity/degradability. Equations are based on first-order kinetics (depending on pool size) where decay-rate constants are controlled by the initial litter quality (mostly represented as CN ratio or recalcitrance) and modified by temperature (t) and humidity (h) (Liski et al., 2005; Parton et al., 1987; Tupek et al., 2019). This representation can adequately be parameterised to simulate a stable soil under unchanging conditions, but cannot explain differences in functioning between soils concerning C and nutrient cycling, plant nutrition and hydrological processes, nor represent changes due to climate, management or pollution (Vereecken et al., 2016). It is also more representative of well-mixed arable lands than of natural soils that have developed horizons, though models have been developed that simulate soil horizons (Aitkenhead et al., 2011).

Recently, research on SOM dynamics has made substantial progress by new conceptual approaches and methodological developments, for example, biogeochemical and physical analyses, molecular and microbial ecology, and novel visualization tools. Vereecken et al. (2016) reviewed key soil processes and existing models, covering different scales and the perspective of a wide range of soil science disciplines. They clearly demonstrate the need to include the contributions of the different ecological compartments involved in SOM dynamics, for example, microbes and fauna, and a revised and more realistic representation of SOM degradability and pools in order to obtain a wider understanding of the soil but they do not include a review of how the soil biota influence the soil.

The role of different functional traits and functional groups of soil biota has been described in the literature. For instance, Schmidt et al. (2011) highlighted the importance of the microbial biomass as key factor in SOM turnover and stabilization and Deckmyn et al. (2014) review the role of mycorrhizal fungi and how to model them. In addition, Filser et al. (2016) argued for the importance of including some representation of soil fauna in soil carbon models. The most important aspect appears to be the activity of ecosystem engineers such as earthworms, ants and termites (see also review by Lavelle et al. (2016)). Soil engineers not only incorporate plant residuels into the soil and mix up soil layers (bioturbation) but also change the soil structure by creating biopores and biostructures (e.g., casts, aggregates) that greatly affect soil hydrology and/or the activity of other soil organisms (Lavelle, 1997). Furthermore, it is also increasingly evident that understanding the complexity of soil food-webs is key to determining the functioning of soil biota and their influence on SOM dynamics (De Vries et al., 2013).

The importance of soil structural modifications on SOM stabilization mediated by soil biota has stimulated the development of models including the explicit representation of structural effects on SOM, which improve predictive capacity without explicit representation of soil fauna (Kuka, Franko & Rühlmann, 2007). Komarov et al. (2017) and Chertov et al. (2017a, 2017b) recently proposed a new mechanistic soil model which incorporates many of these ideas (ROMUL), which however, is quite complex and requires very detailed parameters and measurements.

In this manuscript, we aimed to review the main insights in soil science from different disciplines, with special emphasis on the role of soil biota as a major factor influencing soil C and N dynamics, as well as soil structure and hydrology. We discuss those key processes that can be included in ecosystem models in a mechanistic way. To that end, we review the latest knowledge of key soil processes in terms of chemical SOM concepts, more structurally based concepts, insights into the fine root and mycorrhizal fungal interactions, as well as the key soil faunal actors and how they interact in the soil food web, at a stand-scale. We assess existing models for nutrient (mainly nitrogen, N) and water availability to plants, as well as soil C sequestration and leaching. Finally, we propose a new model concept by extracting the most relevant processes and the minimal community complexity required to understand and predict the overall functioning of the soil concerning C and nutrient cycling and hydrological functioning. Prediction of the faunal food web or microbial biomass is not the goal of this model concept, but a means to improve predictions of soil C and nutrient cycling and hydrology, as well as our understanding of soil functioning in relation to climate change and management.

**Recommended literature:**

Soil health and degradation: Gomiero (2016); Gregory et al. (2015)

Importance of soil fauna: Lavelle et al. (2016); Filser et al. (2016)

Importance of Mycorrhizal fungi: Deckmyn et al. (2014)

Soil modelling: Vereecken et al. (2016)

**Reviewing key pools, processes and existing models**

**Survey methodology**

At present, there are different “schools” for representing SOM turnover and stabilization, with many overlapping views. We review the main concepts from all three main—chemical, physical and biological—aspects to ensure a comprehensive and unbiased approach: (1) The SOM pools-view, depicting SOM pools and their chemical characteristics as the central part of the soil (with structural and microbial effects as secondary determinants), (2) The soil structure view, emphasizing the soil structure and the role of the soil engineers thereon as the main determinant, and (3) The soil food web view, representing soil microbial and faunal food webs and their role in the flow of C and N. Finally, we will discuss the main interactions between SOM, soil structure and soil biota concerning soil aggregation, fate of earthworm casts, structural effects of soil engineers and the important interactions between fine roots, mycorrhizal fungi and SOM. This review was conducted by including leading authors from the different disciplines. Web of Science Core Collection (www.webofknowledge.com), Google Scholar (scholar.google.com), ScienceDirect (www.sciencedirect.com) and ResearchGate (www.researchgate.net) were used to search for manuscripts covering soil models, soil functioning and SOM stabilization/formation but also each of the different soil biota as well as reviews on soil hydrology and soil aggregation.

**Review goal and limitations**

The aim of this extensive review is to identify the key processes and pools involved in soil C, N and water dynamics to form a basis for a new, integrative concept to represent soil in ecosystem models. We foresee a representation with stronger emphasis on mechanistic understanding of soil functioning (in contrast to a more empirical view that describes only the outcome of processes), which can be included in existing models to improve them. Because of the very strict relation between accessibility of SOM, structure and soil water we will also include a review on the soil water modelling.

In terms of nutrient cycling, we focus on nitrogen, because of its obvious link with SOM turnover and because nitrogen data are generally available at ecosystem scale (CN ratios of main pools such as SOM, microbial biomass or plant litter) (Cools et al., 2014; Zhou et al., 2019). Although many of the principles described below for N are relevant for any other element, other mechanisms such as weathering and adsorption/desorption become increasingly important for less mobile elements. Detailed models have been developed to describe these phenomena (Goddéris, Schott & Brantley, 2019; Steefel et al., 2015) but are, at this moment, too complex and require too much detailed input (such as mineral composition, element composition of all soil C-pools), to be applicable at the scale we envisage for our model concept.

**The chemical aspect: SOM pools and turnover**

Soil organic matter is derived from decomposition and transformation of plant (above-and below ground litter) and animal remains (detritus) and organic products (e.g., root exudates). The fate of SOM is primarily determined by a complex interplay of its chemical properties, the composition and activities of soil organisms, abiotic conditions, and different stabilization mechanisms in soil (see review by Stockmann et al. (2013) and Paul (2016)). Due to its mobility, particularly the dissolved form of soil organic matter (DOM) is important for the C and nutrient transport in and between ecosystems and for the contribution to soil forming processes (review: Kalbitz et al., 2000, Kaiser & Kalbitz, 2012). Traditional and more recent perspectives on SOM turnover and its incorporation in SOM models is presented in this and the following section.

**Classic chemical perspective of SOM**

Traditional soil biogeochemical models used in ecosystem models such as RothC (Jenkinson & Rayner, 1977), CENTURY (Parton et al., 1987; Paustian, Parton & Persson, 1992) or Yasso (Liski et al., 2005; Tuomi et al., 2011) define SOM as a number of cascading pools with different intrinsic decomposition rates. Intrinsic decomposition rates can usually be associated with pools having specific chemical and physical properties, and are modified by abiotic parameters such as temperature and moisture (Liski et al., 2005; Dungait et al., 2012). Such models are good at describing the decay of litter and have been well validated with data derived from litter-bag studies (Liski et al., 2005). While pools associated with labile, easy degradable compounds (e.g., sugars) have a fast decay, pools associated with lignified compounds have a slow decay (Taylor, Parkinson & Parsons, 1989; see review by Krishna & Mohan, 2017). Several models assume SOM pools associated with the most recalcitrant compound groups (e.g., humic substances and lignin) and chemically protected (e.g., SOM-clay complexes) account for the long-term stabilization of organic matter in soil (Segoli et al., 2011; Tuomi et al., 2011; Smith et al., 1997).

However, the concept of long-term SOM stabilization due to chemical recalcitrance has increasingly been questioned (Schmidt et al., 2011; Dungait et al., 2012; Cotrufo et al., 2013; Lehmann & Kleber, 2015). There is a growing evidence showing that the formation of stable SOM is largely independent from molecular properties (Kleber et al., 2011; Schmidt et al., 2011; Lehmann & Kleber, 2015). Modern analytical methods could not prove humic substances to be persistent in soil (Schmidt et al., 2011; Lehmann & Kleber, 2015). It rather seems that SOM is a continuum of decomposing substances and even recalcitrant humic compounds can decay rather quickly (Lehmann & Kleber, 2015). In fact, it is increasingly accepted that chemical recalcitrance is primarily important in early stages of litter decomposition (review: Von Lützow et al., 2006, Marschner et al., 2008). Decay rates of plant litter for example, are usually inversely related to their lignin to N ratios, suggesting slow decomposition at high lignin contents (Melillo, Aber & Muratore, 1982; Zhang et al., 2008; Prescott, 2010).

Recent studies have furthermore highlighted that microbial products from the transformation of plant litter rather than plant litter per se are the largest contributors to stable SOM (Mambelli et al., 2011; Cotrufo et al., 2013; Gleixner, 2013).

**Recommended literature:**

Litter decomposition: Krishna & Mohan (2017)

Stability of SOM in relation to ecosystem: Schmidt et al. (2011); Von Lützow et al. (2006)

**The importance of dissolved organic matter (DOM) and nutrients**

Another key element of the SOM dynamics and the soil C and N cycle is the dissolved organic matter (DOM). The incorporation of DOM into microbial biomass is now considered an important pathway of stable SOM formation (Sokol & Bradford, 2019). Most DOM is derived from litter and humus degradation (see review by Kalbitz et al., 2000; Guggenberger & Kaiser, 2003). Recent studies show that while subsurface DOM is linked to recent plant material, DOM in deeper layers consists of older, more processed substrates, mainly derived from microbial turnover (Kaiser & Kalbitz, 2012).

Besides decaying litter and microbial turnover, direct exudation from plant roots (rhizodeposition) can be an important source of DOC in the soil (on average 5% but up to 20% of photosynthates in grassland species) (Pausch & Kuzyakov, 2018)), with very important effects on the surrounding zone. Recent evidence suggests that these belowground DOC inputs may be even more likely to contribute to the formation of stable SOM than litter derived DOM (Sokol & Bradford, 2019).

Because DOM can leach from soils and can move between soil layers, it is important to model DOM separately. A number of models such as LIDEL (Campbell et al., 2016) include the explicit simulation of DOM. A detailed dynamic model (DyDOC) for predicting metabolic transformations of SOM components and the transport and sorption of DOM in different soil horizons with different soil properties was developed and tested by Tipping et al. (2001, 2012), though it does not include soil biology. DOC can be controlled by sorption to minerals and co-precipitation with Fe, Al or Ca, all governed by the soil acidity (Guggenberger & Kaiser, 2003). For this reason, mineral weathering rate should be considered in the models predicting DOC solubility.

In general, the pathways, sorption and desorption processes of the different compounds of DOM and nutrients like phosphorus are extremely complex, and as such hard to include in a simple soil model. There are detailed surface complexation and ion-exchange models which deal with these processes (Weng, Van Riemsdijk & Hiemstra, 2008; Duputel et al., 2013). Models for soil weathering and for adsorption processes that ultimately explain the soluble nutrients available to plants exist, but are complex and require many parameters (for example, PhreeqC, Parkhurst & Appelo, 2013). In Bortier et al. (2018) a relatively simple empirical model within the soil model ANAFORE is used to distinguish adsorbed and soluble P based on pH, without concretely simulating different base cations. Dzotsi et al. (2010) developed a more complex model for P availability that goes beyond the scope of this paper as it requires extensive parameterization. The approaches to phosphorus modelling in ecosystem models have been comprehensive reviewed by Pferdmenges et al. (2020).

Nitrogen is considered to be the most limiting nutrient for soil organisms and besides being part of SOM (and DOM), it is present in soil also in dissolved mineral forms (NH_4_^+^, NO_3_^−^, NO_2_^−^) which are the primary source of nitrogen for plants, but also for the potential N losses via leaching or denitrification. The different approaches to modelling soil N dynamics have been reviewed by Manzoni & Porporato (2009).

Each soil type has an associated a distinct physicochemical environment and development pathway of the soil profile, which affects the chemical composition and stability of SOC in mineral horizons (Rumpel, Eusterhuesa & Kögel-Knabner, 2004; Rumpel & Kögel-Knabner, 2011), by affecting both the living conditions and activity of soil decomposers but also through a distinctive physical and chemical protection. One of the main soil forming processes involved in chemical SOM stabilization especially in deep mineral soils is the “podzolization” which involves a transport of DOM, Al and Fe in solution from the surface to deeper horizons. The process consists of mobilization and immobilization of these compounds (Lundström et al., 2000). General conditions that favor podzolization are the absence of sufficient neutralizing divalent cations due to the presence of parent materials with low amounts of weatherable minerals (Ca^+2^, Mg^+2^), an impeded decomposition of plant litter due to low temperatures and high rainfall conditions that favor the transport of DOC (along with Al/Fe) down the profile (Van Breemen & Buurman, 2002). Moreover, the nutrient-poor status and high acidity typical of this soil type tends to decrease faunal activity which subsequently impedes vertical mixing of the soil and favors vertical differentiation and accumulation of partially decomposed plant residues in organic horizons (Van Breemen et al., 2002; Rumpel, Kögel-Knabner & Bruhn, 2002).

Although few studies have compared C stability among different soil types, some of them suggest that stabilization processes may be soil-type specific and therefore depend on pedogenic processes (Rumpel, Eusterhuesa & Kögel-Knabner, 2004; Rumpel & Kögel-Knabner, 2011). However, representation of pedogenic processes such as weathering or podzolization in mechanistic models is rare (Minasny, Sulaeman & McBratney, 2011) and may be relevant only for longer time scales.

**Recommended literature:**

DOC: Kalbitz et al. (2000)

N mineralization models: Manzoni & Porporato (2009)

**The physical aspect: new perspectives in SOM and water dynamics**

**Structural perspective on accessibility of SOM and diffusivity of water through pore space**

There is a close interaction between soil structure, SOM, water/gas balance, and the size and connectivity of pores as ecological habitats in soil. Recent advances in our understanding of SOM stabilization show that patterns of spatial inaccessibility against decaying soil organisms, or stabilization by interaction with mineral surfaces and metal ions (review: Von Lützow et al., 2006) seem to play a more important role in long term stabilization of SOM than chemical recalcitrance (Dignac et al., 2017; Dungait et al., 2012). These studies show that the main stabilization mechanisms that protect SOM from decomposition are physical protection by soil macro-(250–2,000 µm) and micro-(53–250 µm) aggregate formation and chemical protection associated with silt and clay particles and Fe- and Al- oxides (Dwivedi et al., 2017). The accessibility of SOM to microbes due to pore size and the capacity of microbes to oxidize SOM based on the strength of the organo-mineral associations are two different mechanisms involved in SOM stabilization and SOM dynamics. However, the separation of OM occluded in clay microstructures from “true” organo-mineral associations remains a methodological challenge (Chenu & Plante, 2006; Von Lützow et al., 2008; Yudina & Kuzyakov, 2019). Until this is possible, it might be possible to view organic matter stabilized in organo-mineral associations as in such close contact to the mineral that there is no space for microbes and microbial exoenzymes to physically reach the OM.

It can therefore be argued that the most important mechanism for SOM stabilization over longer time scales is the physical separation of organic compounds from the organisms able to degrade or transform them, for example, in anoxic or dry pore space areas or within aggregates and that this applies also to organo-mineral associations. (see review by Von Lützow et al., 2008). Soil structure and its dynamics are thus the most important factors controlling SOM turnover and sequestration, whereas chemical recalcitrance is only a secondary determinant (Dungait et al., 2012).

Soil structure also determines the soil water dynamics. Water is essential for all soil processes (chemistry, biology, physical transport of DOM and nutrients).

Water availability or water activity in soil is limited by water potential, which in soil is mainly controlled by the adhesion forces to solid particles (matric potential), which, together with the cohesion forces between water molecules, drives capillarity. Water matric potential is considered to be a major controlling factor of SOM turnover and microbial activity (Thomsen et al., 1999). It affects the physiology of microorganisms and many critical mass transfer processes in the pore space: diffusion of soluble organic matter, exoenzymes and gasses, and motility of microbes (see review by Or et al. (2007)). These mass transfer processes can limit microbial access to organic matter at low water contents and, as a consequence, affect its turnover rate (Allison, 2005; Stark & Firestone, 1995). The physical separation of habitats at low water contents is likely what supports the vast diversity of soil microorganisms (Tecon & Or, 2017) as organisms have developed different strategies to mitigate the effect of these barriers (Allison, 2005; Mills, 2003; Torsvik & Ovreas, 2002). In turn, microorganism activities may stabilize (Six et al., 2004) or destabilize aggregates and affect soil porosity (Crawford et al., 2012; Helliwell et al., 2014) or, under extensive microbial growth, may even result in pore clogging (Seki, Miyazaki & Nakano, 1998); providing a feedback to soil structural properties and consequently to SOM turnover. Many soil processes are thus closely interlinked (Six et al., 2004).

**Recommended literature:**

Bacterial activity in soil pores: Or et al. (2007); Tecon & Or (2017)

Stabilisation of SOM through inaccessibility: Von Lützow et al. (2006, 2008)

**Modelling soil structural effects on SOM turnover**

Traditional ecosystem models represent physical and chemical stabilization of C in the soil as an implicit property of the most passive (inert) SOM pool and often relate it to clay content. Although clay content can be seen as a simplified proxy for both SOM stabilization mechanisms (i.e., adsorption and aggregate inclusion) (Rühlmann, 1999, Sulman et al., 2014), it is clearly not their only driver. This has motivated the development of several new models that explicitly account for stabilization mechanisms for effects of either or both mechanisms on SOM turnover (Abramoff et al., 2018; see review by Stockmann et al., 2013; Wieder et al., 2014, 2015; Sulman et al., 2018). For example chemical protection by adsorption onto mineral surfaces is dynamically represented in the COMISSION model (Ahrens et al., 2015). The Struc-C model (inspired by Roth-C), on the other hand, is more aggregate-centric but incorporates both mechanisms by assuming organo-mineral associations are the smallest aggregates and describes the interaction among organic matter and soil structure through the incorporation of aggregation and porosity submodules (Malamoud et al., 2009). Stamati et al. (2013) built on this effort and introduced another Roth-C based model CAST that simulates macro- and micro-aggregate formation and the stabilization of particulate organic matter. Abramoff et al. (2018) proposed to model mineral-associated organic matter (MAOM) and aggregate C as two separate measurable pools but did not actually propose how would they be analytically distinguished. Despite these advances, aggregate formation modelling remains a difficult issue at the stand scale because many of the processes occur at a much smaller scale (Yudina & Kuzyakov, 2019).

A different, more pore-based approach was introduced in the CIPS model (Kuka, Franko & Rühlmann, 2007) which modified the classic empirical SOM pools taking into account soil structure effects. It is based on a quality-driven primary stabilization mechanism (recalcitrance of SOM) and a process-driven secondary stabilization mechanism (site of turnover) of SOM in soil. In addition to the division of SOM into the qualitative pools on the basis of chemical measurability, it takes into account different turnover conditions depending on pore space and accessibility for microbial biomass. The main assumption of the CIPS model is that the biological activity is not evenly distributed through the whole pore space. The pore space classes (i.e., micro-, meso- and macropores) used in the model are marked by wilting point, field capacity and pore volume. Because of the poor aeration in the micropores they show very low biological activity, leading to a strong protection of the C localized in this pore space. This results in the reduction of the turnover activity, related to soil t, h, soil texture, relative air volume and distance to the soil surface. Simulation results show that the bulk density variations have a severe impact on C storage (Kuka, Franko & Rühlmann, 2007). Besides a validation of the CIPS model for longterm experiments representing a wide range of soils and site conditions, (Kuka, Franko & Rühlmann, 2007) show that the conceptual pool of inert SOM (used in many models) can also be described as the amount of C situated in micropores. Consequently this new approach seems more generally applicable than the soil texture based approaches applied so far.

**Recommended literature:**

Modelling soil structural effects on SOM: Kuka, Franko & Rühlmann (2007);

Modelling SOM effects on soil structure: Malamoud et al. (2009);

C sequestration modelling: Stockmann et al. (2013)

**Modelling soil hydrology and structure**

A large number of soil models of varying levels of complexity and dimensionality are now available to describe the basic physical and chemical processes affecting water flow and solute transport in the subsurface environment (Vereecken et al., 2016).

Many models that describe the soil-plant-atmosphere continuum still use simple capacity based soil water flow models to quantify the terms of the water balance (Deckmyn et al., 2008; Farmer, Sivapalan & Jothityangkoon, 2003; Teshima et al., 2006). The main motivation for using these capacity based models is their simple parameterization (Romano, Palladino & Chirico, 2011). They describe water flow in soils as mainly driven by gravitational forces where each soil layer spills over to the lower soil compartment once a critical soil moisture content has been reached (spilling bucket models). This critical soil moisture content is often defined as field capacity and is routinely measured in soil surveys. Soil water storage capacity of a specific compartment can be thus emptied by downward flow, surface runoff, deep drainage, and evapotranspiration processes. Since gravitation is the dominant potential controlling water flow, specific parameterization needs to be included in order to account for capillary rise from a groundwater table into the root zone and lateral flow processes (Guswa, Celia & Rodriguez-Iturbe, 2002). However, this method tends to overestimate soil water in the top layer and underestimate drainage.

More advanced soil models nowadays use Richards equation and the convection-dispersion equation (Jury & Horton, 2004) to describe water and solute movement through soil. Soil models describing water flow based on Richards equation provide more flexibility in incorporating the full complexity of water flow in the soil-plant-atmosphere continuum and its impact on spatially distributed abiotic and biotic processes, including capillary rise, though at a high computational cost (Kuraz, Mayer & Pech, 2014). Many of these processes are characterized by a large spatial and temporal variability with locally distributed hot spots and hot moments. However, these more advanced 3D features are harder to parameterize. To address parameterization difficulties, pedo transfer functions (PTFs) have been developed that allow predicting soil properties and soil parameters that control abiotic and biotic processes. Soil horizons, texture, qualitative structural and morphological information, organic matter content, pH, redox and mineral concentrations are soil properties that can be used in PTFs to quantify soil properties and gain information on functions (e.g., soil hydraulic functions, mineralization constants, sorption properties and ecosystem functions such as providing water and nutrients to plants and regulating biogeochemical cycles) (Bouma, 1989; McBratney et al., 2001; see review by Vereecken et al. (2016) and Van Looy et al. (2017)).

The presence of macropores and other structural heterogeneities can generate flow instabilities and cause preferential flow and transports (review: Jarvis, Koestel & Larsbo, 2016; Beven, 2018; Hendrickx & Flury, 2001). Due to preferential flow, water and solutes may move faster and deeper into the soil profile than what would be predicted by Richards equation, so models using this equation tend to underestimate leaching (Julich, Julich & Feger, 2017; Šimůnek et al., 2009). These macropores are in many cases the consequences of biotic processes, such as earthworms burrowing and root growth (Capowiez, Sammartino & Michel, 2014; Bastardie et al., 2002; review: Wilkinson, Richards & Humphreys, 2009). Modelling approaches for preferential and non-equilibrium flow and transport in the vadose zone were reviewed by Šimůnek et al. (2003). Extensions have been made to consider preferential flow and transport in models based on Richards equation (review: Köhne, Köhne & Simůnek, 2009; Šimůnek et al., 2003). Yet, these models contain several uncertainties due to a lack of observational data at the pore scale and to the inherently dynamic macropore system in soils being subject to physical (swell/shrink, freeze/thaw), biological (variations in soil faunal and microbial activity, root growth, rhizosphere processes) and anthropogenic disturbances (e.g., tillage practices, Capowiez et al., 2009).

Continuous advances in both numerical techniques and computation power are now making it increasingly possible to perform comprehensive simulations of non-equilibrium flow processes in the vadose zone (review: Vereecken et al., 2019). Such simulations, especially if paired with exhaustive field data sets (e.g., by data assimilation), are vital for better understanding and quantifying the effects of heterogeneities, fractures and macropores on flow and transport at the field scale (Van Genuchten, Leij & Wu, 1999; review: Šimůnek et al., 2003).

Challenges in predicting soil water flow and solute transport beyond laboratory scale include: soil parameterization, handling structured soils including preferential flow, handling soil heterogeneity, temporally changing properties (e.g., soil bulk density, structural properties, etc.), and description of root water uptake (e.g., Jury et al., 2011; review: Vereecken et al., 2019). Thus, it is clear that although the importance of soil structure and water are proven, their inclusion in models is hampered because of the lack of data on soil structure and the difficulties in measuring and simulating soil water at the ecosystem scale.

**Recommended literature:**

Preferential flow: Jarvis, Koestel & Larsbo (2016)

Solute transport and preferential flow modeling: Köhne, Köhne & Simůnek (2009); Šimůnek et al. (2003)

Pedotranfer functions: Van Looy et al. (2017)

Infiltration: Vereecken et al. (2019)

Bioturbation: Wilkinson, Richards & Humphreys (2009)

**The biological aspect: the role of the soil food web**

The soil comprises a rich and very diverse community of organisms (Bardgett, Usher & Hopkins, 2005; Bardgett & Van der Putten, 2014; Orgiazzi et al., 2016a; Ramirez et al., 2015). To be able to cope with this high diversity, species can be grouped into functional groups, under the assumption that if species occur at the same location in the soil and share the same resources and predators they should perform the same function (review: Briones, 2014). Research has so far focused on the importance of each one of these functional groups to the ecosystem, but this highly specialised information is not integrated into the more plant-based ecosystem models (Geisen et al., 2019).

It has long been known that litter decay is faster in the presence of macro fauna (comparison between small and larger mesh size litterbags) (reviewed by Frouz et al., 2015). Also, the major roles of soil engineers for bioturbation are well described (see “Soil structural modifications by engineers”), which add to the effect of soil fauna to processes. Recent publications have shown the importance of the diversity of soil organisms in relation to soil functioning and stability, both in the laboratory and in the field (reviewed by Deng (2012); Wagg et al. (2013)). Other studies have shown that an intact soil food web is important for ecosystem functioning because it influences key functions such as decomposition, nutrition retention and nutrient cycling (Bengtsson, Setälä & Zheng, 1996; Philippot et al., 2013). In addition, the soil food web is sensitive to management. Ploughing, soil compaction, litter removal and obviously the use of insecticides are practices that are deleterious to the soil faunal community (Yeates et al., 1997; Wardle et al., 1995), with repercussions for soil processes. Such major negative effects on soil organisms are ignored in the most widely used ecosystem models (ORCHIDEE—Camino-Serrano et al. (2018), PaSim and Biome-BGC—Sándor et al. (2016), ANAFORE—Deckmyn et al. (2008)) that thus cannot realistically simulate these management effects.

**Recommended literature:**

Role of macro-fauna: Frouz et al. (2015)

Role of soil fauna: Briones (2014)

Role of microbial community: Deng (2012)

**Defining and describing food web components**

**Figure 1:**
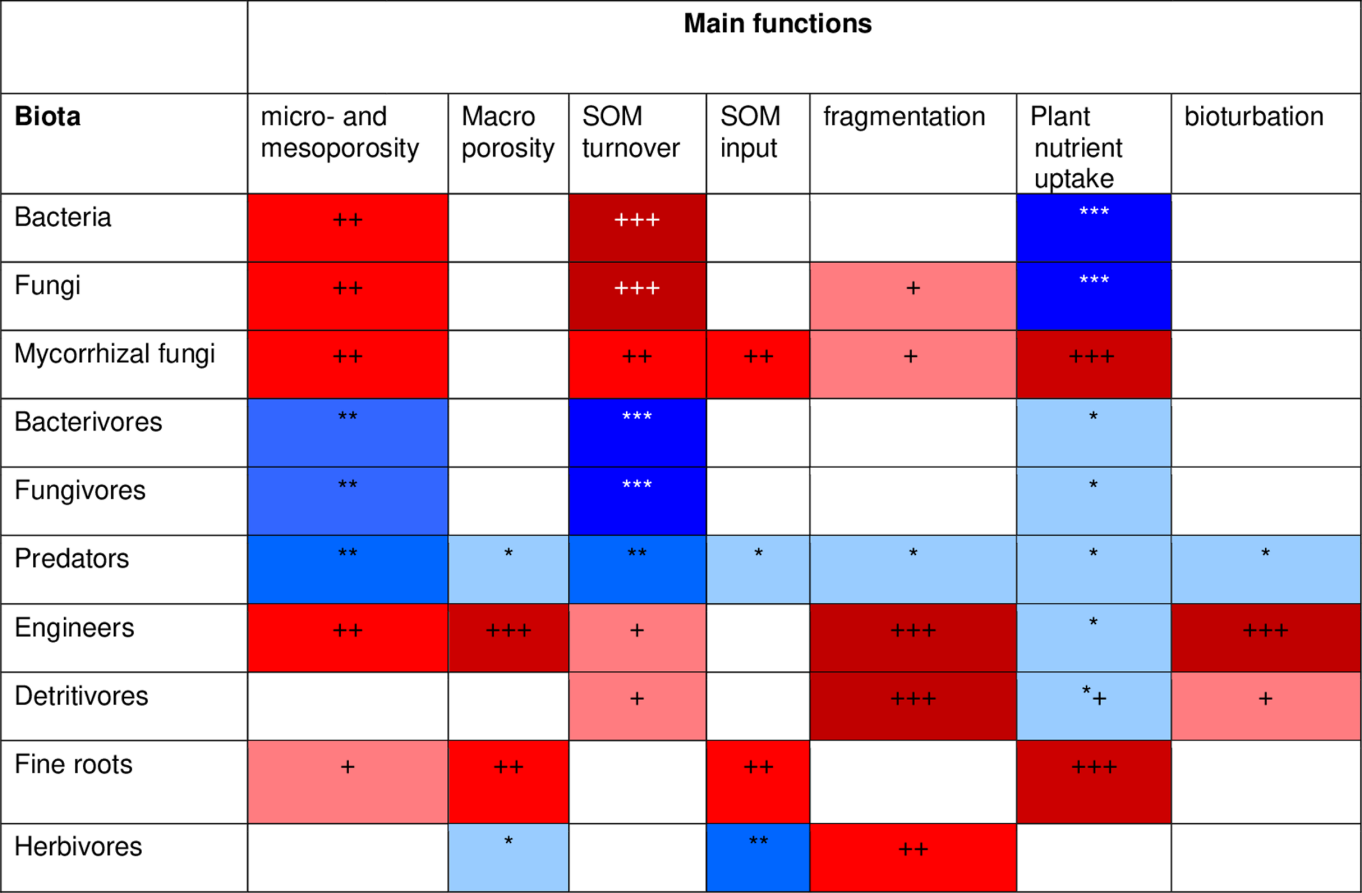
Importance of different functional groups ofsoil biota on key soil processes linked to ecosystem functioning. +, direct effects; *, indirect effects.

To develop a model that simplifies as much as possible the enormous complexity of the soil food web, it is important to review all soil biotic inhabitants and determine keystone species or functional groups within each trophic level. In the following sections, we therefore review the main soil organisms according to their size, trophic level and functional significance, that is, microorganisms (size 1–100 µm), microfauna (<0.1 mm), mesofauna (0.1–2 mm) and macrofauna (>2 mm), as well as fine roots (<2 mm) that are the main primary source of soil C. Simulating larger vertebrate fauna (mice, moles, rabbits, or birds) is beyond the scope of this paper. All size groups of soil fauna include organisms of different trophic level and functional significance. Nevertheless, microbivore soil fauna are usually small-sized members of micro- and mesofauna, whereas ecosystem engineers belong to the macrofauna. In this review we will classify the organisms mainly by function and food source, not by size but we describe for each functional group which organisms belong to it. All biotic effects on the main soil components necessary to simulate SOM as well as nutrient and water flows are described. Figure 1 summarizes how the different functional groups impact on porosity as linked to aggregation (meso-and micropores), macroporosity, SOM turnover, nutrient availability, and C influx into the soil. Since the goal is to understand how to include these organisms in a model we also review, where possible, data on the biomass of the group and of their contribution to the C cycle.

**Soil microorganisms**

The soil microorganisms include bacteria, archaea and fungi which are the primary enzymatic degraders of organic matter (Datta et al., 2017; Johnston, Boddy & Weightman, 2016) and protozoa which are microbivores strongly contributing to the control of the activities of other microorganisms (see review by Gao et al. (2019)). Microbial degradation activity ultimately determines both the rate at which nutrients become available to plants and the amount of C stored in soils (Mambelli et al., 2011; Cotrufo et al., 2013; Gleixner, 2013). Microbes are also known to induce weathering of minerals (Uroz et al., 2009). In this section, we take a functional approach and first discuss bacteria and non-mycorrhizal fungi as significant decomposers and deal with mycorrhizal fungi separately because of their other distinct functions.

**Bacteria and Archaea**

Prokaryotic abundance can vary between 4 and 20 × 10^9^ cells cm^-3^ soil (Bardgett & Van der Putten, 2014). Several studies have shown that at least half of the soil microbial populations are respiratory active (Lennon & Jones, 2011). Bacteria were found to contribute about 35% of the total heterotrophic soil respiration (Joergensen & Wichern, 2008), and their contribution relative to fungi depends mainly on the chemical composition of the SOM.

The classic understanding about the distribution of the microorganisms (especially Bacteria and Archaea) has been that everything is everywhere (Baas Becking, 1934). However, recent studies showed that, in contrast with the classic understanding, bacterial species are biogeographically restricted due to variations in climatic, soil and plant conditions (Bardgett & Van der Putten, 2014). The common view is that there is a high functional redundancy within the soil communities for nutrient mineralization and that changes in community structure rather than changes in species richness play a role in soil and ecosystem functioning (Bardgett & Van der Putten, 2014). For most ecosystem scale purposes however, the classic understanding is adequate.

Bacteria also play a central role in the production and immobilization of inorganic and organic N. Moreover, microbial biomass contributes directly to the pool of soil organic N through its death and turnover (Bottomley, Taylor & Myrold, 2012). It is estimated that about 0.28–28 μg N is assimilated into bacterial biomass (into protein) per g soil and per day (Bottomley, Taylor & Myrold, 2012).

Much of the organic material is degraded by microorganisms carrying out aerobic respiration. When organic matter is transported to zones in the soil where oxygen is low or inexistent, anaerobic respiration can be carried out by specialized bacteria that utilize terminal electron acceptors other than oxygen, such as nitrate, manganese, iron, sulphate or CO_2_ (resulting in methane emissions, the end product of CO_2_ reduction (review: Dalal et al., 2008)). Global methane emissions reach 600 Tg CH4 year^−1^ and it is estimated that water-saturated soils such as peat and rice soils contribute to about 55% of the total methane emissions (see reviews by Le Mer & Roger (2001); Dalal et al. (2008)). This process is however energetically less favorable and comparatively slower than aerobic respiration (Keiluweit et al., 2017). Recent evidence suggests that anaerobic microsites in which anaerobic respiration can take place such as insides of aggregates or peds comprise between 14% and 85% of the pore volume in well-drained soils at moderate moisture (Keiluweit et al., 2018). Mineralization rates in such microsites are reduced by ~90% compared to well-aerated soil compartments (Keiluweit et al., 2017).

Because of their size (0.3–5 µm), bacteria often reside in pores and inner surface of aggregates as micro-colonies of about 2–16 cells (Gupta & Germida, 2015). Higher colonization of bacterial cells is restricted to hot spots with higher available C, such as the rhizosphere or the outer surface of freshly formed aggregate (Foster, 1988). Several studies reported an influence of the physicochemical characteristics (water potential, nutrient and oxygen availability) on the ecology of the bacterial community (Six et al., 2004), which links well with the concepts of the structural availability of SOM as described in “Structural perspective on accessibility of SOM and diffusivity of water through pore space”.

**Non-mycorrhizal fungi**

Fungi, are an important component of soil ecosystem functioning, especially regarding organic matter decomposition (Van der Wal et al., 2013). Their significance lies in their ability to degrade more recalcitrant SOM due to a combination of morphological (hyphal growth form) and physiological (extracellular enzymes) characteristics (Van der Wal et al., 2013).

Fungi can be of two distinct forms: spherical cells (yeasts) or long thread like structures called hyphae or mycelium (filamentous fungi). Filamentous fungi are of particular importance in terrestrial ecosystems as they allow an extended exploration of soil via their hyphal system, penetrating solid substrates (Van der Wal et al., 2013). Hyphae are also very efficient in the translocation of water by bridging air-filled pores (Curiel Yuste et al., 2011) and by bridging nutrient-poor patches by which they supply growth limiting elements to zones of metabolic activity (Frey et al., 2000; Gupta & Germida, 2015). Their average abundance has been estimated at 100 m of hyphal length per gram of soil (Bardgett & Van der Putten, 2014). It has also been estimated that about 1.3–10.9 µg of fungal biomass is formed per g soil per day, corresponding to about 0.06–0.48 µg N immobilized in to fungal biomass (Bottomley, Taylor & Myrold, 2012). Filamentous fungi are fundamental to C decomposition of terrestrial organic matter (review: Geethanjali & Jayashankar, 2016); it has been estimated that fungal respiration can account for up to 65% of the total microbial soil respiration (Joergensen & Wichern, 2008).

**SOM mineralization: bacteria versus non-mycorrhizal fungi**

The ratio of fungal to bacterial biomass is highly variable (between 0.007 and 0.34) among different biomes (Fierer et al., 2009; De Vries et al., 2006). Generally, forest ecosystems have a higher fungal to bacterial (F:B) biomass ratio than grasslands. Fierer et al. (2009) found particularly high F:B in temperate coniferous forest soils, whereas deserts and grasslands had the lowest ratio.

Land-use changes, and agricultural intensification have been shown to shift a fungal-dominated to a bacterial-dominated food web (De Vries et al., 2006). For example, in a study comparing the resistance and resilience of the soil food web to drought, the fungal-based food-web of an extensively managed grassland and the processes of C and N it governs was more resistant to drought than the bacterial-based food web of an intensively managed wheat field (De Vries et al., 2013). Modelling of these two systems revealed that the fungal-based network had a greater evenness that mitigated C and N loss, which made the system more adaptable to drought than the bacterial-based food-web (De Vries et al., 2013).

Through evolution, bacteria and fungi have undergone niche differentiation in the decomposition of organic materials. Fungi have higher biomass C:N, broader enzymatic capabilities, and slower biomass turnover rates (Waring, Averill & Hawkes, 2013). Typically, fungal hyphae are better adapted to nutrient-poor niches in soil than bacteria because they can search for the heterogeneously distributed nutrient resources (review: De Boer et al., 2005). A classic view is that during evolution of terrestrial microbial life, fungi have become specialists in decomposing structurally complex organic matter, such as lignin (recalcitrant litter and SOM), while bacteria, on the other hand, have been able to maintain a significant role in the degradation of simple substrates (review: De Boer et al., 2005). However, fungi and bacteria compete for both complex and simple substrates (Johnston, Boddy & Weightman, 2016), especially for limiting nutrients such as N (Bottomley, Taylor & Myrold, 2012).

As mentioned in the DOM section, plant roots exude substantial amounts (up to 20–40% of their photosynthetically fixed C) of simple and easily degradable organic molecules (see reviews by Badri & Vivanco (2009) and Canarini et al. (2019)). Classically, due to the high abundance of bacteria in the rhizosphere, it was assumed that these easily degradable compounds were almost exclusively degraded by bacteria (Jones, 1998). However, stable isotope probing has revealed that a significant part is also degraded by fungi (Treonis et al., 2004). It has also been shown that fungi are the most active group in the degradation of easily degradable compounds in acid soils (Rousk, Brookes & Bååth, 2009) and at high substrate loading rates, probably due to their superior osmotic stress tolerance (Griffiths et al., 1998).

The degradation of cellulose, the most abundant organic compound on earth (30–50% of plant dry mass), can take place in both aerobic and anaerobic conditions. Aerobic cellulose degradation is widespread within the fungal and bacterial communities (review: De Boer et al., 2005, review: Baldrian & Valášková, 2008). Both aerobic bacteria and fungi produce hydrolytic enzymes, which convert cellulose into glucose (Mansfield & Meder, 2003). Competition for cellulose between fungi and bacteria is high (review: De Boer et al., 2005; Johnston, Boddy & Weightman, 2016). However, it is considered that most of the cellulose is degraded by fungi, because their hyphal growth strategy is better suited to access the cellulose fibres, which are often embedded in a matrix of other structural polymers, such as hemicellulose and lignin (Van der Wal et al., 2013). Contrastingly, in anoxic environments, some bacteria containing cellulosomes that allow enzyme activity to take place directly in their cell are almost exclusively responsible for the cellulose degradation (Lynd et al., 2002).

Lignin degradation is largely, but not exclusively, done by white-rot fungi (Leonowicz et al., 1999) though ligninolytic capabilities have also been reported for Proteobacteria (Bandounas et al., 2011; Tian et al., 2014) and Actinobacteria (Abdel-Hamid, Solbiati & Cann, 2013). The decomposition of lignin needs specialized enzymes (Bödeker et al., 2009; review: Datta et al., 2017) and mostly occurs under aerobic conditions. However, most studies dealing with lignin degradation focus on single strains under laboratory conditions and therefore, a better understanding of lignin degradation and involved C-fluxes through the microbial food web in particular under field conditions is still needed (see review by Datta et al. (2017)).

**Modelling perspectives for non-mycorrhizal fungi and bacteria**

Litter decay rates depend on litter chemistry (e.g., lignin content), but also on microbial activity and the amount of microbial biomass, it is therefore an active process which cannot be adequately represented as depending only on t and humidity of the litter (Deckmyn et al., 2008). It has been long ago proposed to include microbial biomass and activity in soil carbon models (Hunt, 1977), but only the growing recognized importance of microbes in processes such as priming (Neill & Gignoux, 2006) and formation of stable SOM in recent decades has spiked the interest in this idea. This has given rise to a new generation of microbially-explicit biogeochemistry models (reviews Treseder et al., 2012; Sulman et al., 2018) such as MIMICS (Wieder et al., 2014; Wieder et al., 2015), CORPSE (Sulman et al., 2014), LIDEL (Campbell et al., 2016), MEMS (Robertson et al., 2019) and others (Deckmyn et al., 2011; Chertov et al., 2017a). These models explicitly represent the soil microbial community and its role in SOM dynamics; dead microbial biomass is the main contributor to SOM and litter enters the SOM pool primarily via its transformation/incorporation by microbes (Wieder et al., 2014; Wieder et al., 2015; Campbell et al., 2016; Grandy et al., 2016). Microbial activity is modified by temperature and a variable growth efficiency parameter. Microbial biomass is assumed to be at any given time in balance with the available C-sources which is a reasonable assumption.

The modelled effects are usually limited to effects on OM decay/formation and N mineralization, but the important role of bacteria in the N-cycle as denitrifiers or N-fixing bacteria could also be modelled. This might be useful in ecosystems where these processes contribute significant fluxes of nitrogen and closing the N-budget is required (Treseder et al., 2012; Levy-Booth, Prescott & Grayston, 2014). Besides denitrification, modelling other anaerobic processes carried out by bacteria (such as methanogenesis) might be necessary for some ecosystems. In terms of microbial diversity, models have so far mostly distinguished only between two functional groups, be it between fungi and bacteria (Riley et al., 2014), copiotrophs and oligotrophs (Wieder et al., 2014), SOM builders and decomposers (Perveen et al., 2014) or litter-eating r-strategist and SOM-eating K-strategist (Fontaine & Barot, 2005). Bacteria and fungi are often modelled as separate pools because they differ in physiological traits relevant for C and N cycling and their relative abundance influences C and N dynamics at the ecosystem scale (Waring, Averill & Hawkes, 2013; Louis et al., 2016). Even though within bacterial and fungal communities differences exist in terms of life strategies, when compared to each other, heterotrophic aerobic bacteria can be seen as copiotrophic—fast-growing in nutrient-rich environments—, while fungi as oligotrophs—slow-growing, better adapted to nutrient-poor environment (Ho, Di Lonardo & Bodelier, 2017).

Incorporating more detailed information about microbial diversity is controversially discussed (Nannipieri et al., 2003; McGuire & Treseder, 2010; review: Nielsen et al., 2011; Graham et al., 2014). The diversity of soil microorganisms (e.g., species richness and relative contribution of each species to the community composition), is vast, with a high level of functional redundancy in C and N transformations, which makes it difficult to explicitly integrate the microbial diversity in soil C and N models (Louis et al., 2016). Although including only two or three (if mycorrhizal and non-mycorrhizal fungi are distinguished) functional groups of microbes substantially underrepresents their observed functional diversity in soils, the use of multiple SOM decomposing microbial functional groups has not been explored to date (review: De Graaff et al., 2015).

In our view, in many cases it can be enough to distinguish between fungi and bacteria assuming the former are more oligotrophic and the latter copiotrophic. This approach is practical because of their differential contribution to SOM decay, and also because F:B ratio can be easily related to soil C:N ratio and/or to pH similar to the approach in Romul_Hum (Chertov et al., 2017a, 2017b; Fierer et al., 2009).

Based on the very fast life cycle of bacteria, and the ‘everything is everywhere’ hypothesis that states that when conditions change the bacterial community will change as well, the bacterial community can for example switch quickly to an anaerobic life style. Building predictive models that link dynamically changing microbial communities to ecosystem function by explicitly calculating population dynamics is probably less necessary/relevant at the time scales interesting for ecosystem studies (Widder et al., 2016; review: Succurro & Ebenhöh, 2018).

**Mycorrhizal fungi**

Mycorrhizal fungi are a group of soil dwelling fungi that form a symbiotic relationship with a vast majority of vascular plants (Brundrett & Tedersoo, 2018). Mycorrhizal fungi provide host plants with nutrients and improve biotic and abiotic stress tolerance (see review by Smith, Anderson & Smith (2015) and Pozo et al. (2015)), often leading to increased plant diversity and productivity of the host plants (Van der Heijden, Bardgett & Van Straalen, 2008; Van der Heijden et al., 2015; Tedersoo, Bahram & Zobel, 2020). Mycorrhizal fungi require C from their host plants to grow and form hyphae (mycelium) extending into the soil to take up water and nutrients (mainly N and P) that are subsequently transferred to their plant hosts (Smith & Read, 2008). While the nutrient to C exchange rates are highly variable, plants trade 15–30% of their C for gaining on average ca. 75% of their required N; for the fungi, this represents all of their required C at a cost of 40% of their N (Hobbie & Hobbie, 2006; Smith & Read, 2008). The C transfer from the plant to the mycorrhizal hyphae can occur quickly, contributing up to 30% of the total respiration in soil (Söderström & Read, 1987).

Structurally, there are several different types of mycorrhizal interactions (mycorrhizas). The most common types are the ectomycorrhizas (EcM fungi) with high number of taxa and a low number of plant partners but dominant in many ecosystems; arbuscular mycorrhizas (AM fungi) with a low number of taxa but a high number of plant partners and ericoid (ErM fungi) and orchid mycorrhizas (OrM fungi), which are restricted to plants in the Ericaceae and Orchidaceae families, respectively (review: Frąc et al., 2018). The C flux from plants to AM mycorrhiza has been estimated to 5 Pg per year (Bago, Pfeffer & Shachar-Hill, 2000) which represents about 10% of global NPP (50–60 Pg, Nemani et al., 2003). If we compare it with the 5% of NPP allocated to rhizodeposition (Pausch & Kuzyakov, 2018) this suggests that AM fungi receive a significant proportion of the belowground labile C inputs. In one gram of forest soil, tens to hundreds (50–800) of meters of EcM mycelia can be found, representing 20–30% of the total soil microbial biomass (Söderström, 1979; Leake et al., 2004, review: Ekblad et al., 2013). Mycelial biomass corresponding to EcM fungi can range from 100 to 600 kg ha^−1^ (Wallander, Goransson & Rosengren, 2004; Cairney, 2012; Hendricks et al., 2016) or up to 1.5 Pg of AM fungal biomass globally (Treseder & Cross, 2006). Mycorrhizal fungi also contribute to soil structure and aggregation (Lehmann & Rillig, 2015) and their senescing hyphae provide C to the soil (Wilson et al., 2009). They also play a role in water absorption and transport (Johnson et al., 2012) even between multiple trees or seedlings (Warren et al., 2008).

For the plants, AM fungi are thought to be more important for uptake of P and mineral or other readily available N, whereas some EcM and ErM fungi are able to break down SOM to obtain nutrients, mainly N (Moore et al., 2015; De Vries & Caruso, 2016) but also P (review: Tedersoo & Bahram, 2019). Thus, mycorrhizal fungi can play key roles in mobilizing organic N trapped in the SOM for plant primary production (Rineau et al., 2013; Shah et al., 2016). The EcM fungal mycelium can retain in its biomass a high proportion of N (Lindahl et al., 2007) which can prevent up to 50% of nitrate leaching losses; reductions of organic N and P leaching have also been reported. The uptake and immobilization of N by EcM fungi may also aggravate and stabilize a state of strong N limitation in nutrient-poor forests (Näsholm et al., 2013; Franklin et al., 2014). It has also been proposed that EcM fungi compete with the decomposer community for organic N and restrain activities of saprotrophs (Bödeker et al., 2016). This is known as the Gadgil effect (Fernandez & Kennedy, 2016) and results in a decrease of the nutrient content of SOM, reduced SOM decomposition and an increase in soil C (Orwin et al., 2011; Averill, Turner & Finzi, 2014; Averill, 2016).

**Modelling perspectives for mycorrhizal fungi**

EcM and AM fungi form the most common types of mycorrhizas and it is therefore reasonable to include them in general soil/ecological models (Treseder, 2016). Several models have been developed to include the mycorrhizal symbiosis in individual plant models (reviewed by Deckmyn et al. (2014)), but they are rarely included in ecosystem models. Examples of such ecosystem models are the MoBilE and MYCOFON models (Meyer et al., 2010; Meyer, Grote & Butterbach-Bahl, 2012), the C accumulation model MySCaN (Orwin et al., 2011), an AM fungal distribution model proposed by Schnepf & Roose (2006), the mycorrhiza C partitioning model described by Staddon (1998), and the EcM forest model by Franklin et al. (2014). These models represent the symbiotic trade of C and mineral nutrients between plants and fungi, which is modelled in different ways. The most parsimonious approach is based on the assumption that fungi only transfer N that is taken up in excess of their own N demands to the plants (Näsholm et al., 2013; Franklin et al., 2014).

Recently De Vries & Caruso (2016) have developed a conceptual model for the soil food web considering the ability of EcM fungi to decompose SOM by extracellular enzymes (Read & Perez-Moreno, 2003; Phillips, Ward & Jones, 2014), previously only attributed to non-mycorrhizal fungi. Using a mechanistic model, Baskaran et al. (2017) showed that the capacity of EcM to decompose SOM leads to reduced soil C, increased tree growth and a shift in the balance between microbial groups.

In summary, while the key role of mycorrhizal fungi in providing nutrients to plants in exchange for C is relatively well understood, this is not true for the effects of mycorrhizal fungi on SOM decomposition. Because of the global importance of mycorrhizal symbiosis and the large C and nutrient fluxes involved, more research on these effects are urgently needed. As far as the uptake of nutrients is concerned, it is not unrealistic to simulate mycorrhizal fungi as ‘part’ of the plant fine roots. However, the main drawback is that only mineral N and P can be taken up by the plant, whereas in reality mycorrhizal fungi can also obtain nutrients from recalcitrant SOM and thus play a vital role in the SOM dynamics (Deckmyn et al., 2014).

**Recommended literature:**

Root exudates and microbes: Badri & Vivanco (2009);

Enzymatic degradation of lignin: Datta et al. (2017);

Fungi versus bacteria: De Boer et al. (2005);

Importance of diversity of soil organisms: De Graaff et al. (2015);

Protists: Gao et al. (2019);

Fungal decomposition: Geethanjali & Jayashankar (2016);

Mycorrhizal fungi: Smith, Anderson & Smith (2015); Deckmyn et al. (2014); Tedersoo & Bahram (2019)

Modelling of microbial systems: Succurro & Ebenhöh (2018)

**Microbivores**

Microbivores are animals that feed on the soil microflora (i.e., bacteria, Archea and fungi). In terms of size, they belong to both microfauna (protists, nematodes) and mesofauna (mites, collembolans, enchytraeids). Microbivores exert a primary control on bacterial and fungal biomass and activity, with cascading effects on soil carbon and nutrients (Bardgett & Van der Putten, 2014; Blanc et al., 2006; review: Trap et al., 2016; review: Gao et al., 2019). For example, a recent review revealed that, although on average, the presence of active bacterivores reduces soil microbial biomass by 16%, they increase soil respiration by 29%, plant biomass by 27%, and shoot N and P contents by 59% and 38%, respectively (Trap et al., 2016). In other words, the flow of C and N through soil, and possibly other elements, from the bacterial and fungal pools to the SOM pool and to plants is controlled by the size, activity and efficiency of microbivores (Berg et al., 2001; review: Frouz, 2018). Therefore, proper simulation of their effects in a food web SOM model is most likely crucial.

Microbivores are generally divided into bacterial and fungal feeders. Bacterial feeding organisms are generally small (mostly microfauna) and include notably nematodes such as Cephalobidae and free-living protozoans such as Amoebae and Flagellates (Blanc et al., 2006). Fungal feeders include families of nematodes which use a stylet or spear to penetrate fungal hyphae of saprotrophic or mycorrhizal fungi (Yeates et al., 1993). Mites and collembolans (mesofauna) are also important grazers of bacteria and fungi, but not exclusively so, as they also consume other food sources such as plant litter (Brussaard, 1997). In general, larger animals will tend to ingest plant litter and soil together with microbes. Not so many data are available concerning their abundance. Pausch et al. (2016), using ^13^С labelling, found 51 mg C m^−2^ in bacterial feeders and 68 mg C m^−2^ in fungal feeders in an arable maize field.

Although microbivores have probably little direct impact on soil structure (Lehmann, Zheng & Rilliga, 2017), the opposite is not true, as soil structure is thought to have a large influence on the predation potential of microbivores. For example, Cephalobidae nematodes have a much higher impact on bacterial community composition and biomass in large pores than in the bulk soil, presumably because they cannot access pores smaller than 10 μm (Blanc et al., 2006). Likewise, microbial biomass and diversity is highest in microaggregates while nematode abundance and diversity is highest in large macroaggregates (Zhang et al., 2013). It is therefore likely that changes in soil structure with both SOM content and activities of soil fauna engineers induce a feedback mechanism on microbivores.

As far as DOM is concerned, there are several studies showing that microbivore soil fauna can increase the rate of N leaching (Setälä et al., 1990; Toyota et al., 2013; Williams & Griffiths, 1989). Similarly, Liao, Xu & Zhu (2015) compared litterbags accessible and not accessible to microbivores and found that microbivores decreased the CN ratio in DOM. One possible explanation is that faunal grazing can reduce microbial immobilization of N (Carrera et al., 2011). This change in CN ratio of DOM can affect the rate of decomposition in the soil.

**Modelling perspectives for microbivores**

Microbivore functions in soils should be taken into consideration in our efforts to improve SOM models for predicting soil fertility and C sequestration. Many of the needed parameters have been evaluated for some organisms, but the number of studies is still too limited to reliably quantify the overall effect of microbivores on ecosystem functioning (Trap et al., 2016). Nonetheless, initial values from these studies might be enough to start exploring their effects on soil C, N and P dynamics. Predicting microbivore effects in specific environments remains difficult (Trap et al., 2016) but a first effort targeting generic simulation of effects would be of great value.

The diversity of soil fauna feeding on the microorganisms and, at least for some of them, the non-specificity of their diet pose two challenges in terms of modelling. First, it is not clear if a common parameterization can be used for one generic pool of microbivores. For example, do fungal and bacterial feeders have a similar CN ratio, respiratory quotient, generation time and mortality rate? Although it is certainly not the case, standard parameters across a wide spectrum of organisms should be investigated. For example, microbivore composition has been reported to affect neither trophic-level biomass nor the response to increased resource availability (Mikola, 1998). The second challenge is that larger soil fauna, that is, mesofauna, do not feed exclusively on the soil microflora but might also digest litter, thereby creating an overlap between potential model pools of detritivores, on the one hand, and microbivores, on the other hand. The modelling concept based on nutrient stoichiometry (see Predators) developed by Osler & Sommerkorn (2007) is also relevant for microbivore microorganisms as well as for larger soil faunal predators.

It is clear that microbivores require more attention in our studies, so their role can be better understood and represented in more detail in SOM models if proven as beneficial for their predictive power. Given the current, limited, data, they can be simulated as a link between the microbial biomass and the larger predators and detritivores. Because these links and their importance in terms of SOM fluxes, are largely determined by pore size distribution, we would suggest to simulate only the micro faunal microbivores in simple models.

**Recommended literature:**

Protists: Gao et al. (2019)

Soil macro-and mesofauna effects on decomposition: Frouz (2018)

Bacterivores: Trap et al. (2016)

**Predators**

Soil predators are represented in each size class of soil fauna (micro-, meso- and macrofauna) and include predatory protists, nematodes, mites, centipedes, and others. The three size classes also form a hierarchy where larger animals prey on smaller animals as well as on prey of their own size. For instance, the main microfauna groups, nematodes and protists, have predators preying within and among them including Protozoa feeding on nematodes and vice-versa (Geisen, 2016). Isotopic studies have demonstrated that predators form a soil fauna group of their own, that is, an isotopic niche (Korobushkin, Gongalsky & Tiunov, 2014), including spiders, Gamasida and nematodes, preying on microbivores, detritivores and herbivores. Even the neanurid collembolans are classified as predators (Hopkin, 1997; Potapov et al., 2016), thus inhabiting the same isotopic niche as the afore mentioned predators.

Predation in soil challenges our conception of a boundary between aboveground and belowground biota. Aboveground predators, such as spiders, beetles and harvestmen in fact feed on prey traditionally considered to be soil organisms.

While predatory mites, spiders and beetles are ubiquitous, centipedes are rare in conventional agricultural systems, but enjoy the conditions offered in biological agriculture. One of the consequences seems to be that under conventional agriculture there sometimes is a higher impact of pest species (herbivores) because of the lack of predators (Kladivko, 2001).

Soil predators can influence the entire food web by creating important secondary effects. For example, bacterivorous nematodes have been shown to increase plant P uptake by different mechanisms. Nematode predators can decrease bacterial grazing and thus increase mineralization by bacteria, because of the higher bacterial turnover (Ferris et al., 1998) They can also have a hormonal effect on plant roots increasing branching and therefore P-uptake capacity of the plants (Ranoarisoa et al., 2018).

**Modelling perspectives for predators**

To our knowledge, there are no ecosystem models that include soil faunal predators, apart from the Romul-Hum extension to the ROMUL model (Chertov et al., 2017a, 2017b). In this model, predators are not a dynamic pool but a fixed part of the soil food web depending on soil characteristics. It is clear that more data are necessary to validate the population dynamics of predators and subsequently their effect on SOM dynamics. However, as suggested above, some important effects of differences in management cannot be simulated without including the predators.

The model framework described by Osler & Sommerkorn (2007) shows how using nutrient stoichiometry could be an effective and simple way to include the influence of predation on the C and N cycling. The main concept of their framework is that soil fauna with a high C-efficiency and prey with a similar CN ratio contribute to the mineral N, while inefficient assimilators that consume prey with a higher CN ratio would contribute more to the DOM pool.

Given the larger size and longer life-spans of many predators, simulating their effects as “in balance” with the environment seems unrealistic. To model the effects of land use changes (e.g., agricultural conversions, tillage, etc.) or drought periods/flooding in a more realistic fashion including a dynamic pool of predators seems a worthwhile extension to existing ecosystem models for many environments.

**Recommended literature:**

ROMUL-Hum model: Chertov et al. (2017a, 2017b)

CN ratio’s through soil faunal network: Osler & Sommerkorn (2007)

**Herbivores**

Herbivores eat living plant material, such as leaves, flowers, stems and roots. Herbivores exert an influential role in plant community dynamics (Bever, 2003), which in turn determines the amount and quality of plant litter entering the soil and the density and tissue quality of roots. Herbivores have an effect on the amount of SOM via different actions. About 50% of net primary production occurs belowground, in the form of roots, while the largest part of aboveground primary production enters the soil in the form of litter (Sinacore et al., 2017). Root herbivory affects plants in all ecosystems (see meta-analysis by Zvereva & Kozlov (2012)). Andersen (1987) reported up to 30% of root biomass is lost through herbivory. Accordingly, the invertebrates with a greater effect on carbon dynamics are root feeders (Treonis et al., 2001).

Root herbivores are a diverse soil fauna feeding group. Among microfauna they are represented by the plant-feeding and plant parasitic nematodes. These feed mainly on plant juices by tapping into the root. The density of plant-feeding nematodes varies greatly among ecosystems, but due to their short life cycle and fast reproduction they can significantly affect plant communities, including a severe reduction in the crop yields (Yeates et al., 1993). Symphyla and prostigmatid mites (partly) belong to the mesofauna and are also considered root feeders (Striganova, 1980; Orgiazzi et al., 2016b). However, the most influential root herbivores in terms of effects on the plants they feed on are found in the macrofauna, and include Diptera larvae (mainly midges), caterpillars and some major groups of beetles, such as click beetles and curculionids (mainly their larvae) (review: Johnson & Rasmann, 2015).

The highest recorded average density of Symphyla (plant-feeding Myriapoda) is around 10.8 × 10^3^ m^−2^ (Belfield, 1956). The few other sources generally report lower densities, around 200 ind. m^−2^. With an average individual dry weight of 81 µg, this translates in an annual mean biomass estimate of 58 mg m^−2^ (Reichle, 1977). Prostigmatid mites can be very abundant in temperate coniferous forest (up to 2 × 10^5^ ind. m^−2^; 30 mg dry weight m^−2^), and less abundant in tundra systems (around 10^3^–10^4^ ind. m^−2^; 10 mg dry weight m^−2^, Petersen, 1982), with a mixed oak forest in between (Lebrun, 1971). An average dry weight of about 0.5 µg (range 0.2–4.0 µg) is assumed in most data sets, resulting in an average biomass ranging between 10 mg m^−2^ (tundra and temperate deciduous forest) and 50 mg m^−2^ in tropical grasslands (Petersen, 1982).

Diptera larvae are probably the most important meso- and macrofauna root herbivores in terms of the overall effects on plant growth and physiologyy (but there are also caterpillars, wireworms, weevils and other insect herbivores), (Stevens et al., 2000, Samson, 2001). Their average biomass ranges between 10 mg dry weight m^−2^ in tropical grasslands to 0.47 g m^−2^ in tundra ecosystems (Petersen, 1982). Being of larger size, beetle densities will on average be much lower than Diptera densities. Based on average biomass estimations for predaceous beetles (Carabidae and Staphylinidae), that is, ranging between 10 mg m^−2^ to 0.12 g m^−2^ (Petersen, 1982), the biomass of root feeding beetles (Elateridae and Curculionidae) is probably in the same range (Parker & Howard, 2001).

**Modelling perspectives for root herbivores**

The number of studies on consequences of root herbivore-plant interactions for the ecosystem is still quite limited (Andersen, 1987; Eissenstat et al., 2000, see also “Fine roots”). However, the available information from many studies on specific plant-root herbivore interactions (Zvereva & Kozlov, 2012) is enough to start exploring the effects of introducing root herbivores in SOM models on soil C and nutrient dynamics. Predicting root herbivore effects in a specific environment remains difficult, due to a number of often unknown factors, that is, species composition, actual density, ecological efficiencies (which can deviate considerably between modes of feeding), and population turn-over rates or generation times, but a first effort targeting generic simulation of effects would still be of great value. At an ecosystem level fine root turnover is one of the most important C-sinks, and the fate of fine roots (whether they die or are eaten) could potentially have a major effect on the simulated C-balance (Brunner et al., 2013).

**Recommended literature:**

Root longevity: Eissenstat et al. (2000)

Root herbivory: Zvereva & Kozlov (2012)

Root-feeding insects: Johnson & Rasmann (2015)

**Detritivores**

**Mesofauna detritivores**

Mesofauna detritivores feeding on decomposing organic matter (plant and animal remains), also called saprophages, include enchytraeids, collembolans, large groups of mites, some small-sized Diptera larvae, Protura and Diplura. The first three groups have been recognized as having major ecological importance in terms of abundance and biomass whereas the rest have been subjected to very little specific research and will not be further included. As a whole, their primary role shifts between promoting physical (fragmentation) or chemical changes of the organic material ingested, depending on the group of species (Wallwork, 1970, review Dervash et al., 2018). These transformations mainly occur at the top layers (organic soil horizons but also in the litter layer, under stones, etc.) due to their limited burrowing abilities. As explained earlier (see “Microbivores”) apart from ingesting litter, mesofaunal detritivores also graze on bacteria and fungi and thus belong also to microbivores.

**Enchytraeids**

General population density estimates range from 10,000 to 300,000 individuals m^−2^ (O’Connor, 1967; Briones, Ineson & Heinemeyer, 2007), with the majority occupying the upper layers (the 0–4 cm can concentrate >70% of the total population; Briones, Ineson & Piearce, 1997). The main factors controlling their population sizes and vertical distribution are temperature and moisture.

There are no quantitative reliable estimates of enchytraeids’ consumption and digestion rates or agreement on their preferred food sources. As a rule of thumb, it is believed that they feed on organic matter (20% of their diet), bacteria (40%) and fungi (40%) (Didden, 1993). Like earthworms they burrow through the soil and ingest the soil. More recently, C dating techniques performed on field populations have established that they feed on 5–10 year old organic matter (Briones & Ineson, 2002). Importantly, temperature-driven increases in their population size results in a greater competition and thus, when biomass reaches a value of 2.1 gm^−2^ (Briones, Ineson & Heinemeyer, 2007) consumption of older organic matter substrates increases and consequently, also a greater release of non-labile C occurs (Briones, Ostle & Garnett, 2007). Interestingly, in certain ecosystems, such as coniferous moder soils their metabolic contribution has been estimated to be 11% (O’Connor, 1967) and is comparable to that exhibited by woodland earthworm populations (8–10%; Satchell, 1967).

**Collembolans**

Collembolans are important as epigeic decomposers (Ponge, 1991) and metabolic rates are comparable to those for enchytraeids and nematodes of similar weight (Mc Millan and Healey, 1971).

As many as 53,000 m^−2^ (equivalent to 330 mg m^−2^) have been found in a limestone grassland (Hale, 1966). However, their numbers fluctuate seasonally and with food availability, and for example, 670,000 individuals m^−2^ have been recorded in permanent moist soil in Antarctica covered by the alga *Prasiola crispa* (Collins, Baker & Tilbrook, 1975). Predation seems to be the primary regulatory factor of their population sizes (Wallwork, 1970).

As many hexapods, they accumulate a high proportion of fat in their bodies (54% of dry weight or 24% of live weight) which increases with age (Anderson & Healey, 1972). Importantly, they shed their exoskeleton several times as they grow (up to 60 times in their lives) and in exuvia representing 2–3% of body weight (Anderson & Healey, 1972) which could be an important source of nutrients for other soil organisms.

**Oribatid mites**

Although the majority of oribatid mites are considered to be panphytophages (Luxton, 1972), more recent work (Schneider et al., 2004) indicated that besides fungal feeders and predators, there are larger groups that can be defined as primary and secondary decomposers and hence, having a preference for litter at different decomposition stages as well as being coprophagous (feeding on fecal material) (Petersen & Luxton, 1982). Their role in soil mixing is small compared to other invertebrates but they play an important role in humus formation and mineral turnover (Hoy et al., 2008). They produce fecal pellets, which help to distribute organic matter and are prone to microbial attack. Mites can colonise all soil horizons, including the mineral layers and can reach up to 3 × 10^5^ ind. m^−2^ in temperate mixed forests (Petersen & Luxton, 1982). These high densities are the result of their fast life cycles, which in the case of small species could be several generations per year (Mitchell, 1977).

**Macrofauna detritivores**

Macrofauna detritivores include soil organisms that are larger than 2 mm, such as isopods, millipedes, earthworms, ants, and termites. They either live in litter or excavate the soil in search for plant remains and SOM. The engineering capacities (burrowing and bioturbation) of some species in this group will be discussed further (see “Soil structural modifications by engineers”), but they also have an important role in the C-cycle.

Macrofauna detritivores can reach very high densities and biomasses. For example, earthworms are abundant as long as the climate is humid and warm enough, at least for a certain part of the year. When soils contain enough organic matter (for endogeic earthworms that ingest soil and digest SOM) and primary production is high enough (for epigeic and anecic earthworms that eat plant litter) earthworms can be very abundant (i.e., more than 10^6^ individuals ha^−1^) and their biomass can be as high as 1,000 kg ha^−1^ (Lavelle & Spain, 2001). Endogeic earthworms may ingest more than their own weight of soil each day, so depending on their abundance and climate they may process all the soil in 5 years or less (review: Curry & Schmidt, 2006).

**Quantitative contribution of detritivores to SOM transformations**

The bulk of plant-derived C enters the soil only when the vegetation dies. A fraction of it is transformed by the decomposers through breaking down the organic substrates and assimilated into their tissues, another fraction is released as fecal material and/or exuvia, respired as CO_2_ and finally deposited as dead bodies (Petersen & Luxton, 1982).

There are very few estimates of how much organic material is ingested, digested, assimilated and respired by individual groups. In one year, detritivores (including earthworms) may consume 20–100% of the total annual input of litter (Frouz et al., 2015). Certain species, such as blanket bog enchytraeids are responsible for processing 40% of the total litter input (Standen, 1973).

Even fewer attempts have been made to measure how much of the ingested organic matter has been assimilated. Overall, it has been suggested that the range of assimilation efficiencies is wide (1–65%), with oligochaetes being the least efficient (Petersen & Luxton, 1982). Under laboratory conditions, the measured metabolic activity of enchytraeids and collembolans per unit of dried weight seems to be twice that of oribatid mites (compiled by Wallwork, 1970). In certain ecosystems where these organisms are dominant, their contribution could have a great influence. For example, in moorland soils, 70–75% of the total energy is assimilated by the dominant enchytraeids (Heal, Jones & Whittaker, 1975).

A certain amount of energy ingested is metabolized and most of it is dissipated in respiration. Temperature has a strong influence on soil respiratory metabolism. For example, in a laboratory incubation of a grassland soil, Q_10_ significantly increased and was 25% greater in the presence of enchytraeids (Q_10_ = 3.4) than in their absence (Q_10_ = 2.6) (Briones, Poskitt & Ostle, 2004) and even higher values were reported when enchytraeids were incubated in a peatland soil (Q_10_ = 3.9; Carrera et al., 2009).

In the field, the whole picture gets complicated because estimates change with population densities (and hence, with biomass and age structure) that are known to fluctuate with seasons (and thus, with variations in ambient temperature and moisture conditions).

A good quantitative assessment was provided by Petersen & Luxton (1982), who concluded that soil detritivores are reasonably efficient in assimilating organic matter (40–50%) and have a community growth efficiency of 10–20%; 4–-85% of the assimilated energy is dissipated in respiration, with only 15–50% being allocated to growth and reproduction. In addition, coprophagy is important since it allows a better reutilization of organic substrates that were not fully digested on first consumption.

Furthermore, the role of soil animals on the retention of other nutrients can also be crucial: McBrayer (1977) estimated that 70% of the N released during litter decomposition is immobilized by soil invertebrates. Similarly, MacLean (1980) indicated that up to 1 mg P and 10 mg N m^−2^ are found in dipteran adults emerging from tundra soils forming a major redistribution mechanism in these nutrient-poor soils.

On the other hand, detritivores can also increase the mobilization of C, N and P. For example, enchytraeids have been found to release significant amounts of CO_2_ and dissolved organic C (DOC) (Briones, Carreira & Ineson, 1998; Briones, Poskitt & Ostle, 2004; Carrera et al., 2009, 2011). They also increase leaching of dissolved organic N (DON), ammonium and phosphorus (Briones, Ineson & Poskitt, 1998). Similarly, significant increases in the leaching of ammonium, nitrate and calcium occurred as a consequence of collembolan grazing (Ineson, Leonard & Anderson, 1982).

**Modelling perspectives for detritivores**

Mesofauna detritivores have not been included into ecosystem scale models so far, and information at this scale is scarce. Nonetheless, their impact on the ecosystem has been shown to be significant (Frouz et al., 2015; Filser et al., 2016).

In our view, it is not possible (for lack of data) nor necessary for a simple SOM model (with the goal of modelling soil ecosystem functioning and C, not soil fauna) to distinguish the different mesofauna detritivores. However, parameterization of the saprotroph pool can mimic the differences between them. In the simplest case, this can be seen as a fixed relative abundance of the various species that determines the ‘average’ parameters. Besides maximal growth rate and respiration, CN ratio and response to temperature (Q_10_) are important to characterize this group, as is the production of excrements, exuvia and exoskeletons that need not be addressed separately but can be an important flux. From the review it seems clear that distinguishing only between C used for growth and C respired is not an adequate representation. Although the concept of recalcitrance has been questioned, it can still be used here to allow some chemical changes by detritivores that slow down decay and favor fungal decay above bacterial decay.

For macrofauna detritivores quite a number of models have been developed that often focus on their engineering capacity, these models are discussed in “Soil structural modifications by engineers”.

**Recommended literature:**

Soil meso-fauna effects on SOM and litter: Dervash et al. (2018);

Enchytraeids: Briones & Ineson (2002)

**Fine roots**

The rhizosphere, the area of soils conformed by the fine roots and the microorganisms directly associated with them, has been shown to be of great importance to soil C and nutrient dynamics (Adamczyk et al., 2019; Kriiska et al., 2019). Fine root dynamics and activity includes the production of biomass and necromass as well as a continuous release of exudates from roots that is the base food for a large community of soil microorganisms and soil fauna (e.g., detritivores, herbivores) (Juan-Ovejero et al., 2020). Nowadays, the definition of ‘fine roots’ is under discussion, as the commonly used 2-mm threshold (Finér et al., 2007) is not a functional criterion and lumps together both thin and woody transport roots and absorptive roots with primary structure (Ostonen et al., 2017). Despite the fact that thin woody roots, absorptive roots and associated mycorrhizal mycelium cycle carbon at significantly different rates (Godbold et al., 2006; Leppälammi-Kujansuu et al., 2014), fine root turnover is a significant and dynamic C sink. Furthermore, the direct input of DOC from fine roots is important for leaching and for all interactions with soil biota (Juan-Ovejero et al., 2020).

The root litter contribution to soil C is often underestimated (Rasse, Rumpel & Dignac, 2005). Live roots contain high concentrations of soluble and easily decomposable organic substrates (e.g., glucose, malate, cellulose, peptides such as glutamate), whereas root necromass is rich in organic constituents (lignin, suberins) characterized by lower decomposition rates (recalcitrant substances) (Rasse, Rumpel & Dignac, 2005). The composition of the roots is considered to be relatively similar to the above-ground parts, showing a similar pattern between deciduous (higher in nutrients and soluble compounds) and coniferous (higher in lignin and liposoluble compounds) species although this relationship has not been found within species (Hobbie et al., 2010). However, differences in fine root activity (production and mortality) and decomposition among ecosystem types are not well known (Coleman & Hendrix, 2000) and even less is known regarding the impact of species on the amount and composition of root exudates though it has been shown the impact on the ecosystem can be significant (Yin, Wheeler & Phillips, 2014). Root and hyphal exudates particularly rich in readily available constituents may induce a small but significant increase in litter decomposition indicating an active role of the rhizosphere in soil priming (Grayston, Vaughan & Jones, 1997; Kuzyakov, Friedel & Stahr, 2000; Rasse, Rumpel & Dignac, 2005). Moreover, root turnover can be increased by 50% by grazing (Eissenstat et al., 2000) as described in “Herbivores”.

**Modelling perspectives for fine roots**

In many ecosystem models, fine roots are still simulated as a single pool with a single turnover rate. Though some data on fine root distribution are available (Fan et al., 2016; Finér et al., 2011) this is not the case in the majority of ecosystem studies since fine root measurements are time-consuming. Furthermore, when root growth is not well defined over the soil layers, nutrient and water uptake is obviously not simulated realistically over the layers as well. The rate and description of fine root turnover in models (constant proportion of NPP, or following the seasonality of leaf area (constant leaf to fine root ratio—Deckmyn et al. (2008)) has a major effect on the model outcome in terms of total C budget (McCormack et al., 2015). Fine root production partially follows aboveground productivity (Abramoff & Finzi, 2015) but is not yet fully understood nor implemented in models.

To link fine root growth with soil hydrology (water and nutrient uptake), a more detailed approach is required. Novel root architecture models and tomography techniques have facilitated the development of three-dimensional functional-structural models as reviewed by Dunbabin et al. (2013). The description of root water uptake has been advanced through more complex approaches that explicitly describe water flow in both the soil and inside the root system (Javaux et al., 2008; Schröder et al., 2009; Mai et al., 2019). Yet the impact of specific rhizosphere hydraulic properties on the root water uptake at the plant scale is generally not considered, except for instance in Schwartz, Carminati & Javaux (2016). Models that simulate root growth and nutrient uptake processes, like R-SWMS or OpenSimRoot, enable calculation of nutrient uptake as the roots grow and receive photosynthates from the shoot (Postma et al., 2017). Examples of coupling of the root growth model RootBox with soil models are presented for example, in Schnepf, Leitner & Klepsch (2012) who simulated root system phosphate uptake from a rhizotron as affected by root exudation. In most of those models, root architecture is used to compute volumetric sink terms for water or nutrient uptake. Few examples exist that explicitly simulate the roots as physical objects with uptake prescribed via the boundary conditions at the root surfaces (Leitner et al., 2010; Daly et al., 2017; Schnepf et al., 2020).

However, these improved descriptions are not yet sufficiently incorporated into larger scale models (Hinsinger et al., 2011; Vereecken et al., 2016). Recent initiatives in this way already include soil resistance, plant root distribution and climatic demand, to upscale to the macroscale (Javaux et al., 2013). There remains an overall lack of spatially explicit models that properly describe soil C and nutrient dynamics at different spatial scales (Manzoni & Porporato, 2009).

How macropores are used by roots and how roots create macropores or induce compaction are still challenging questions (Lesturgez, Poss & Hartmann, 2004) which only start to be included in models (Landl et al., 2017).

**Modelling soil food webs**

Soil food web modelling has mainly been used to calculate the flow of C and nutrients through soil and to investigate the role of the various functional groups in these flows (Scheunemann et al., 2016; Malard et al., 2020). This kind of modelling requires knowledge about the architecture of the food web (“who eats who”), the biomass of the functional groups and physiological information, such as generation time, growth and death rates and metabolic efficiencies (see chapters above for details). The importance of these types of models in explaining N and C cycling was already shown in the late 80’s and 90’s (Hunt et al., 1987; De Ruiter, Neutel & Moore, 1994; Berg et al., 2001); however, this knowledge did not find its way into the basically plant-centred ecosystem models and relatively little advance in the domain has been made. Nonetheless, Berg et al. (2001) and Schröter, Wolters & De Ruiter (2003) used such food web models at a forest ecosystem scale to show the importance of functional groups for predicting C and N dynamics in the soil.

To model the C and nutrient fluxes, many food web models first calculate the feeding rates among the functional groups. Next, C and N mineralization are derived from the feeding rates of functional groups using metabolic efficiencies, that is, assimilation and production efficiencies, and CN ratios of consumer and resource. The equations used to calculate the feeding rates follow the approach of “inverse modelling”, which goes back to O’Neill (1969) based on the conservation of matter and energy and the assumption that the system is at steady-state. This approach has first been applied to soil food webs by Hunt et al. (1987) and later by De Ruiter, Neutel & Moore (1994), Berg et al. (2001) and Schröter, Wolters & De Ruiter (2003).

Alternatively to a steady-state description, different approaches exist for modelling the growth of a species population within a food web, more often applied to insect populations (see review by Ju & Shen (2005)). The first approach is to simulate an increase in population towards the carrying capacity of the system. This yields stable and reliable results, but does not allow for a strong influence of management or climate on the carrying capacity, so it is not so different from assuming a steady state. Other models opt for a more Richards’ shaped growth curve, where growth rate goes to a maximum, allowing a direct link between resource and species and a dynamic representation of climate and management effects. To be sensitive to climate change a daily time step is most appropriate at a stand scale. Daily faunal pool sizes can be calculated as a set of linear equations for each pool including growth, turnover and respiration. A dynamic representation of all populations is thus possible. We have found no models using such an approach at an ecosystem scale however, although current computational power should allow this. The new ROMUL model (Chertov et al., 2017a, 2017b) has a detailed representation of soil fauna in 15 groups. This is the first model (to our knowledge) simulating the effect of the faunal food web, including necromass and respiration, on the C and N cycle of a soil. The biota are assumed to be at steady state and climate and management affect them only empirically.

**Interactions between SOM, soil structure and soil biota**

The processes involved in SOM stabilization are strongly controlled by soil biota. Bacteria and fungi are considered to be the most important soil microorganisms involved in the formation and stabilization of aggregates, especially at the microscale (Gupta & Germida, 2015, review: Costa, Raaijmakers & Kuramae, 2018), though there is still considerable debate (Lehmann, Zheng & Rilliga, 2017; Oades & Waters, 1991; Six et al., 2004). In fact, mycorrhizal fungi are known to influence the movement of SOM into mineral soil (Frouz et al., 2001, Ponge, 2003) but also the formation and stabilization of aggregates. Ectomycorrhizal fungi affect soil aggregation (reviewed in Rillig & Mummey, 2006) through changes in the root architecture by (1) covering fine roots with fungal mantles (Smith & Read, 2008), (2) producing hydrophobins in the mycelium and rhizomorphs (Tagu et al., 2001; Mankel, Krause & Kothe, 2002) that help adherence to different soil surfaces, (3) enmeshing and entangling soil primary particles, organic materials and small aggregates, and (4) oxidizing of biomolecules present in SOM that leads to the formation of aggregates of organic matter (Kleber & Johnson, 2010; Kleber et al., 2015). In sandy soil, only hyphal networks are able to tie the abundant sand particles to form stable aggregates (Six et al., 2004).

Bacteria can also have a profound influence on soil aggregation (Six et al., 2004). Like fungi, bacteria produce exopolysaccharides, which act as glue and help organic residues to attach to clays, sands and other organic material, resulting in the formation of new microaggregates (review: Degens, 1997). In addition, other groups of soil biota, such as microarthropods, are assumed to affect SOM stabilization; most likely by influencing organo-mineral interactions (e.g., by effects on soil chemistry and leachate) and aggregate formation (e.g., by necromass, eggs as aggregate starting point) (Maaß, Caruso & Rillig, 2015; Soong & Nielsen, 2016). Similarly, it has been shown that earthworms can play a central role in physical stabilization of newly generated organic matter through soil aggregate formation (Pulleman et al., 2005; Rillig & Mummey, 2006; Six & Paustian, 2014; Bottinelli et al., 2015; Angst et al., 2017, 2019) during cast formation (see below).

The link between aggregation and porosity is hard to quantify. Regelink et al. (2015) showed for different soils that overall soil porosity is the sum of the structural porosity (determined by clay, sand and silt fractions) and aggregation. They concluded that micropores (which they define <9 µm) are mainly situated within the aggregates, while mesopores are situated between dry-sieved aggregates. Total porosity increased with total aggregate content, and the fraction of micropores increased with increasing dry-sieved aggregate content. In this study, macropores were not studied but obviously, biopores are also part of the soil porosity.

**Recommended literature:**

Microbial effects on aggregation: Costa, Raaijmakers & Kuramae (2018);

Mycorrhizal effects on soil structure: Rillig & Mummey (2006)

**Feaces**

When macrofauna is present, a substantial part of litter is turned into macrofauna excrements that are either holo-organic (such as millipede fecal pellets) or in the form of organo-mineral aggregates (such as earthworm casts) (Frouz & Kuraz, 2013). They can be deposited in the soil or at the surface in large quantities and in the case of some species of earthworms the surface aggregations of intact and fragmented litter together with defecated soil around the openings of the earthworm burrows are called “middens” and represent important microhabitats for microbial activities (Brown, 1995; Orazova, Semenova & Tiunov, 2003).

Several authors have shown that microbial activity increases during and shortly after faunal feeding but then decreases and may be lower in faunal faeces than in the non-ingested litter (Frouz & Šimek, 2009; Frouz, Santruckova & Elhottova, 1999; Lavelle & Martin, 1992; Tiunov & Scheu, 2000). The increase in microbial activity in fresh faeces is often attributed to litter fragmentation (Gunnarsson, Sundin & Tunlid, 1988; Kaneda et al., 2013) which increases surface area and may thereby increase microbial access to the litter. Artificial litter fragmentation experiments have shown, however, that litter fragmentation alone may both enhance or suppress microbial activity (Gunnarsson, Sundin & Tunlid, 1988; Kaneda et al., 2013).

The reasons for the decrease in decomposition rate and hence in the stabilization of SOM in the older faeces of soil fauna are also variable. Some macrofauna species, such as earthworms, consume organic matter together with soil mineral particles (Schulmann & Tiunov, 1999; review: Curry & Schmidt, 2006). This results in the binding of SOM in aggregates, which may slow decomposition and help stabilize SOM (Gunina & Kuzyakov, 2014; Lavelle, 1988; Six et al., 2004). In the case of macrofauna that mainly consumes litter without soil, the reduced decomposability of their faeces is associated with changes in their chemistry compared to that of the original litter. The faeces are usually depleted in easily available polysaccharides, degraded by invertebrate enzymes (Frouz, Novakova & Jones, 2002), and are enriched in lignin (Frouz et al., 2015; Hopkins, Wheatley & Robinson, 1998). Because the easily available substances are not present in faeces, the decomposition rate is reduced (McInerney, Little & Bolger, 2001; Bossuyt, Six & Hendrix, 2005). The content of soluble phenols decreases after passage through the gut of litter-feeding fauna (Coulis et al., 2009; Frouz et al., 2015; Špaldoňová & Frouz, 2014), which may be caused by precipitation with proteins, making phenols insoluble (Frouz et al., 2015) but at the same time also reduce N availability. Although earthworms are typically the main group contributing to faunal-mediated aggregation (Marashi & Scullion, 2003), faecal pellets produced by micro-arthropods have also been recognized as important contributors to aggregate formation (Maaß, Caruso & Rillig, 2015), either by promoting porosity or by filling the pore space between particles and hence, impairing fungal growth and decomposition.

For earthworm casts at the surface, aggregate degradation by rain can have a significant impact on their stability and the subsequent leaching of nutrients (Decaëns et al., 1999) and similar effects have been found for termite mounds (review: Jouquet et al., 2011).

**Recommended literature:**

Earthworms: Curry & Schmidt (2006);

Macro-aggregation: Degens (1997)

Termites: Jouquet et al. (2011)

**Soil structural modifications by engineers**

By definition, ecosystem engineers are organisms that have a measurable impact on the physical properties of their environment either through their activities or their mere presence (Jones, Lawton & Shachack, 1994; review: Jiménez & Lal, 2006).

Such organisms are thus often very influential for the functioning of ecosystems and tend to affect all organisms and their activities with which they share a common environment (Lavelle et al., 2016). Note that engineers are also important because they can create heterogeneity in physical, chemical and biological features at various spatial scales (Barot et al., 2007; Jiménez, Decaëns & Rossi, 2012; Jouquet et al., 2007; Raynaud, Jones & Barot, 2013) and likely strongly influence the functioning of food webs (Sanders et al., 2014). Three concurrent and interrelated processes are behind the engineering capacity of soil organisms but are generally considered separately for practical reasons: (i) biopore formation, (ii) bioturbation (soil mixing) and, (iii) fauna-mediated aggregation (see above “Feaces”).

**Biopore formation**

Many soil organisms can be considered as ecosystem engineers and are very influential for soil processes (Lavelle, Bignell & Lepage, 1997; Lavelle et al., 2007). Indeed, soil biota require space and connectivity between pores to move through the soil, to forage for nutrients and/or carbon-based energy sources, water and living space (e.g., plant large roots and macrofauna such as earthworms, ants or termites). This can be achieved either by pushing aside soil aggregates or by ingesting soil (e.g., in earthworms), creating the so-called biopores that remain after roots death or the passage of fauna (Schneider et al., 2018).

Some soil macrofauna is particularly influential for soil structure through their engineering activities, such as ants (review: Folgarait, 1998), termites (Dangerfield, McCarthy & Ellery, 1998) and earthworms (Lavelle, 1988, Lavelle et al., 2007). As an example, values between 0.013 and 0.024 m³ earthworm burrows m^−3^ of soil have been reported (Bastardie, Capowiez & Cluzeau, 2005), that can persist for very long periods in the soil.

**Bioturbation**

By burrowing through the soil and dragging litter, soil engineers mix mineral and organic materials from the different horizons in a process known as bioturbation. The extent and type of bioturbation largely depend on the ecological behaviour, body size and population density of the different species, and earthworms are a good example to illustrate this. Earthworms are traditionally classified into three main ecological groupings (Bouché, 1977; Brown, 1995): epigeic, endogeic and anecic species. Epigeic and anecic earthworms consume fresh litter at the soil surface, whereas endogeic earthworms ingest more mineral soil creating a network of galleries and soil aggregates of various sizes (earthworm casts). While epigeics and endogeics mostly move horizontally in their respective layers, anecic earthworms create permanent or semi-permanent vertical galleries. Therefore, the latter group plays a more important role in mixing the soil and incorporating litter into the soil profile. Taken together, earthworms are thus very influential for soil structure (Blanchart et al., 1999) and subsequently for water drainage, aggregate stability, mineralization and leaching of mineral nutrients (Edwards et al., 1989; Jouquet et al., 2008; Lavelle et al., 2020).

It is generally considered that bioturbation tends to stabilize SOM by promoting physical protection (see Filser et al., 2016), although the deep burial of litter or casts is an often overlooked mechanism that could significantly contribute to carbon persistence in soils, also favoured by the more stable conditions (Špaldoňová & Frouz, 2014). However, some authors have highlighted that in some systems, wetter conditions in the deeper layers might accelerate SOM turnover (Rasse et al., 2006). To elucidate this, more information is needed regarding the decomposition rates of buried casts and C sequestration processes in earthworm burrow walls (Zhang et al., 2013).

Similarly, ants and termites build nests by gathering different organic and mineral materials, creating SOM hotspots. This creates soil physical and chemical heterogeneity (Lovegrove, 1989; Jouquet et al., 2002; Dean, Milton & Klotz, 1999). Little is known of the horizontal transportation carried out by termites during the construction of their fungus-growing chambers or those by ants with their anthills. Both ants and termites bring food to their nests (which are locally partially returned to the soil as faeces) and create fungal gardens in some chambers so that these nests often constitute patches enriched in organic matter and mineral nutrients (Folgarait, 1998; Dangerfield, McCarthy & Ellery, 1998).

In agroecosystems, plant residues are artificially incorporated in soil by tillage but in natural ecosystems, besides bioturbation by fauna, the processes incorporating those materials into the soil are rather limited (i.e., soil flooding and consequent burial by mud, burial by mineral particles brought by wind or water erosion, or cryoturbation). This is why, when macrofauna is absent, litter mostly accumulates at the soil surface, and can only reach deep soil after its physical fragmentation into small pieces and washing down by percolating water (Bohlen et al., 2004; Hale et al., 2005). Hence, faunal activity determines to a large extent if organic matter and processes such as decomposition mostly happens on the soil surface or in deeper soil horizons, and thus affects the amount and quality of organic matter incorporated into the soil.

**Soil engineer models**

Most models on soil engineers focus on the effect of earthworms on mineral soils. Some models only tackle the demography of earthworms or their movements (Martin & Lavelle, 1992; Klok, Van der Holt & Bodt, 2006; Pelosi et al., 2008; Vorpahl, Moenickes & Richter, 2009), to predict their impact on soil functioning. Other models such as the Multi Agent System model, SWORM, simulate the movements of individual earthworms within a soil profile and the consequences for soil structure (Blanchart et al., 2009). Barot et al. (2007) modeled at a larger scale (about 100 m^2^) the feedbacks between earthworm demography and soil aggregates. Another analytical model (Barot, Rossi & Lavelle, 2007) allows predicting the impact of earthworm on mineral nutrient stocks and primary production from the impact of earthworms on fluxes of mineral nutrients within the ecosystems and losses of nutrients from the ecosystem (e.g., through leaching). More recently, a simulation model was developed to predict the impact of an invasive earthworm on the dynamics of soil C taking into account earthworm effects on microorganisms (Huang et al., 2010). In the future, this model may help predicting the speed of earthworm invasion. The activities of anecic earthworms are incorporated in the ROMUL_Hum model (Komarov et al., 2017; Chertov et al. (2017a, 2017b). There are few models tackling the impact of other soil engineers such as ants or termites on soils, except for the work by Dangerfield, McCarthy & Ellery (1998) on termites.

**Recommended literature:**

Ants: Folgarait (1998);

Soil fauna effects on bioturbation and aggregation: Jiménez & Lal (2006);

**Model concept**

**KEYLINK general concepts**

The goal of this effort is to integrate the current views on the central role of soil biota in SOM and soil water dynamics into a mechanistic model. The challenge faced was to minimize model complexity while retaining enough detail to predict and analyse effects of changes in climate and management of a very wide range of soils (grasslands, forest, agricultural soil, organic and more mineral soils) including the key processes and the key species according to the most recent insights.

**Figure 2:**
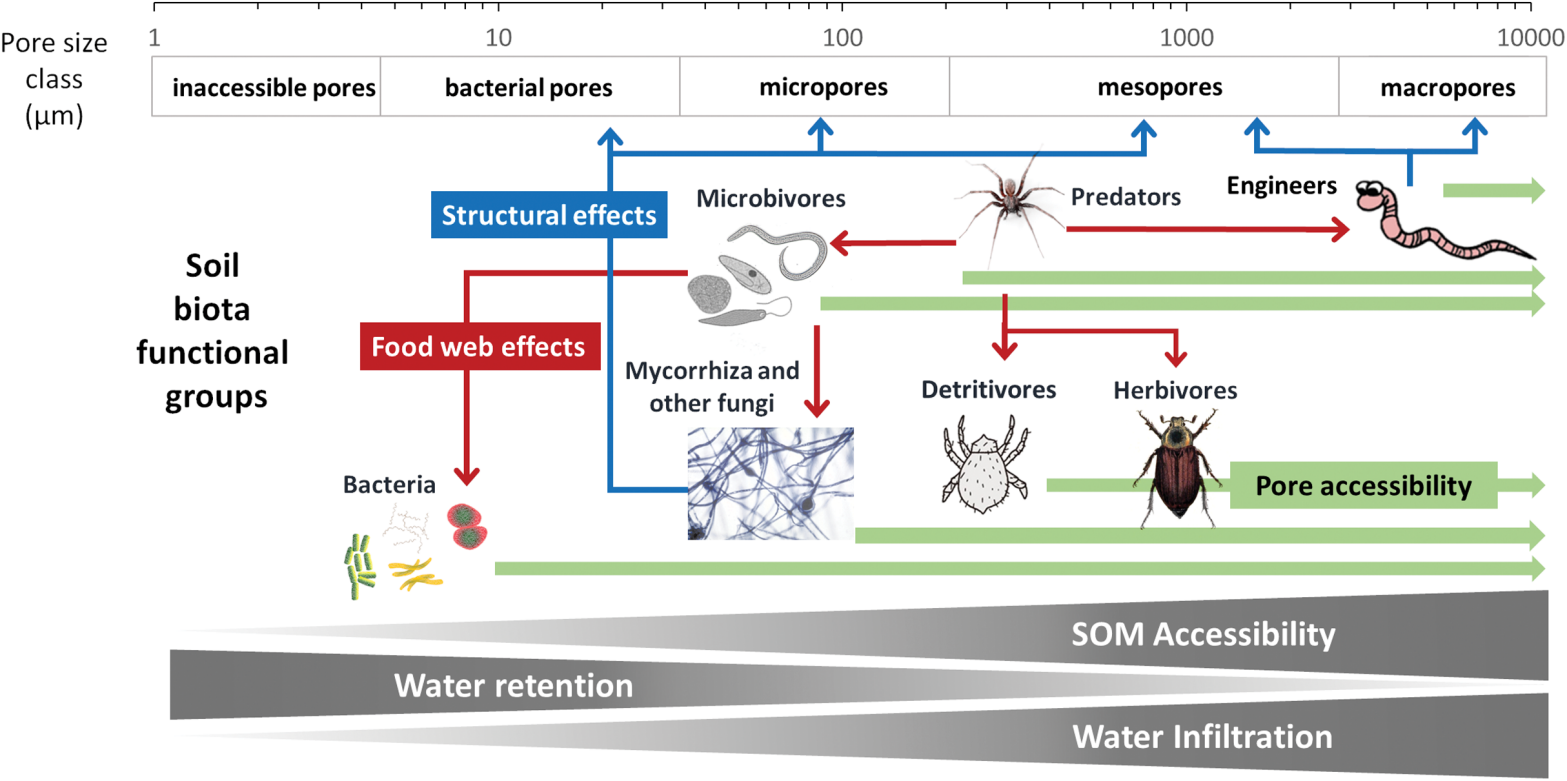
The KEYLINK model representation of the soilbiota functional groups and their relationship with soil structure. Pore size distribution is represented by five classes. Blue arrows in­di­ca­te effects on soil structure; green arrows indicate the range of pore class­es accessible to each group; red arrows indicate trophic effects by grazing and preda­tion. Grey triangles show the ex­pec­ted responses of water re­ten­tion, in­fil­tra­tion and SOM stabilization. Image credits: spider by Kratochvil (2019), CC0; bacteria by Fondren (https://pixy.org/4793065/), CC0; mite by unknown (https://pixy.org/2176637/), CC0, protists by Klapper (2012), CC0, herbivores by Hochdanz (1876), CC0, fungi by CDC & Georg (2012), CC0. Earthworm drawn by co-author (OV).

From our extensive review our main conclusion is that placing chemical recalcitrance at the center of a soil model is not the best representation of soil functioning. Instead we propose soil structure as the central part of our soil model, since structure determines ‘accessibility’ for the dynamic soil faunal pools in terms of pore sizes and body sizes of soil fauna (Fig 2), but also the hydrological properties (soil water flow) and the associated temperature flow of a soil. Our key assumptions are:

- Litter and SOM decomposition are active processes, conducted by microbes and soil fauna and thus dependent on the consumer pool size.
- Decomposition depends on accessibility (function of pore size distribution and the related local soil water content and aeration) and secondly on the quality of the decomposing material.
- Pore size distribution determines the accessibility to all soil biota, but also the hydrology and the availability of O_2._
- Soil water flow depends on soil pore distribution which is also a function of the activity of soil engineers and aggregation by soil biota.
- In soils where soil engineers are important (most mineral soils), it is essential to simulate their effect on bio pore formation and bioturbation, for some organic soils their effect is less important.
- Mycorrhizal fungi need to be represented in the model regarding their interaction with the plant (important input of C to the soil), decay of SOM and effect on soil aggregation.
- In many cases a real food web, with dynamic faunal and microbial pools is necessary for example, to simulate management or climate change effects. The diversity and number of trophic levels changes with soil types/ecosystems. When there are not enough data however, and when changes are slow (stable situation) a representation with constant pools of soil fauna can be considered.
- Special attention needs to be paid to the simulation of fine root turnover which should either include herbivory or herbivory should be simulated.
- Modelling aggregation in detail is beyond the scope of an ecosystem model, the most important effects of aggregation can be included through the concept of the pores (aggregation increasing micropore fraction and reducing mesopore fraction) as influenced by engineers (casts), bacteria and fungi.

To use the KEYLINK concept, a good hydrology model with multiple soil layers is necessary. For soils where, besides the water availability, distinct horizons are present with very different characteristics, each horizon should be simulated separately, but in other cases it can be adequate to use layers only for the hydrological calculations.

**KEYLINK soil structure representation**

We define different pore sizes, based on measurability and accessibility by soil fauna as well as hydrological concepts. The initial pore size distribution can be calculated from water retention measurements.

Soil structure is dynamic: it can be modified by engineers, by aggregation (by bacteria and fungi which glue soil particles together), by organo-mineral interactions (function of clay content and SOM), but also by precipitation (destroying macropores and aggregates) and management (increasing bulk density). In a multi-layer soil system, bioturbation by soil engineers can be a major factor.

**Description of SOM pools**

Concerning size and the main decomposing biota, a distinction between larger particulate material (fresh litter, fragments, and necromass) and SOM is required. Within SOM dissolved DOM and particulate POM need to be simulated separately to allow leaching, but can be simulated as in balance with each other. Mineral associated C is not a separate pool but a fraction of SOM depending on porosity/clay content.

Fungi and bacteria have different capabilities to decay litter. Therefore, we need to add enough description of the initial litter quality. The average recalcitrance (defined here as % non-hydrolysable compounds) and CN ratio are enough for a main division between these three pathways. SOM need not be further divided into pools. However, SOM is distributed across the pore space and depending on the pore size distribution, it is more or less accessible to decomposers. Accessibility is defined by pore size distribution by calculating the surface area of each pore fraction at each time step, and distributing the soil SOM across this area.

**Soil biota**

We opted for a minimal complexity with which we can still include the best understood faunal and food web effects, the important distinction between the bacterial and fungal pathway as well as the potential feedback effects of management in reducing food web complexity. The main division is based on function, not family or size.

- Non-mycorrhizal fungi
- Bacteria
- Mycorrhizal fungi
- Fungivores and bacterivores (or total microbivores)
- Predators
- Root herbivores
- Detritivores (non-engineers)
- Engineer detritivores

The different roles of all biota are summarized in Fig. 1. Engineers are part of the food web, and in addition create biopores and casts (changing accessibility by reducing pore size within the cast), and bioturbate the soil.

**Figure 3:**
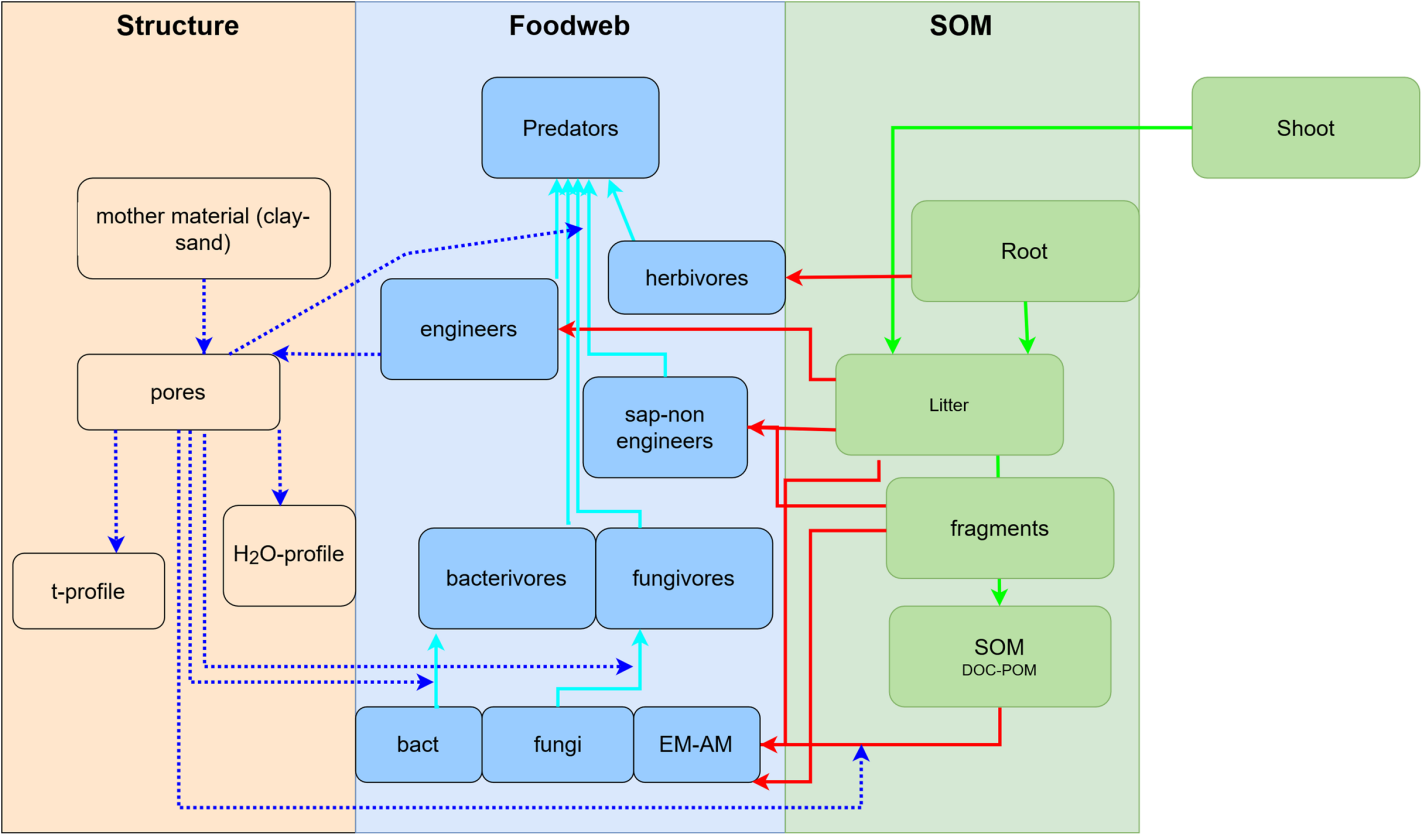
KEYLINK model concept. Full arrows, fluxes; dotted arrows, influences.

In our view, the most simple soil model can ignore all changes in chemistry apart from the initial litter quality, and decay is calculated from pore size distribution and environmental parameters (in combination with consumer pool size) (Negassa et al., 2015; Strong et al., 2004; Toosi et al., 2017). However, for a more complete model, all biota can change “recalcitrance” and CN ratio of the material they consume by producing faeces that are more stable. All biota respire and become necromass that enters the SOM. The interaction between the biota is shown in Fig. 3. Since the goal is to simulate the response of the soil functioning to climate and management, the soil fauna need to be responsive to both. We suggest calculating the faunal pools as a set of linear equations with the change in the pool size dependent on growth, respiration (depending on t), faeces (including exoskeletons), and turnover (natural death and predation). Growth can be calculated as a function of maximal growth rate, resource availability (as a function of pore sizes) and quality, and environmental parameters (t and pH). The CN ratio and sensitivity to pH and t, as well as respiration rates and fecal production need to be included for each biota.

**Model application and parameterisation**

The KEYLINK model has been implemented (Flores et al., 2020, unpublished data) and data are available to allow its development. However, full validation of the concept requires some crucial data which are missing in many experiments. For example, many ecosystem studies do not include soil fauna data at all, or only the diversity but not the abundance or biomass. While earthworms have been quite intensively studied the effect of termites and ants on soil C dynamics is less known. On the other hand, experiments focusing on soil fauna often do not include crucial data concerning the ecosystem such as litter quantity and quality, and fine root biomass and turnover. Soil structure and hydrology are very seldom described in detail, in many cases limited to sand and clay content and bulk density. Concerning hydrology, preferential flow through biopores is seldom taken into account. For a better representation of N availability, models on nitrifying/nitrogen-fixing bacteria would be necessary. For many other nutrients (including P), representation of the mineral weathering and the adsorption/desorption including a dynamic pH model would be required but in many cases data are lacking to parameterise such models. To evaluate our concept, data from isotope studies could be of great value, especially if they include the faunal food web as well as the microbial composition, the fine roots and the mycorrhizal fungi.

This very general model concept should be parameterized and implemented differently according to the specific ecosystem but will allow comparison across these different systems (which is not possible using most current models that focus on specific ecosystems (ANAFORE—Deckmyn et al. (2008), Franklin et al. (2014)) or grasslands (PaSim—Sándor et al. (2016)).

In organic soils, a focus on chemical decomposition can yield adequate results if the different pathways are included in an active way (microbes divided between bacteria, fungi and mycorrhizal fungi with different characteristics and efficiencies for transforming different food sources). For such soils, it is important to know at least the CN ratio and the ‘recalcitrance’ and to include the interaction between mycorrhizal fungi and plants. Inclusion of faunal effects (the composition will depend on C content and hence pH) and improved hydrological description (requiring structural description of the soil) should be able to improve the modelling results. For very wet soils (e.g., peatlands), it is clear that a correct distinction between anaerobic and aerobic processes should be included.

In the case of mineral or organo-mineral soils, the incorporation of pore-size distribution in the mineral layers will better describe the (in)accessibility of SOM due to physical inaccessibility (only bacteria can access the smallest pores, and they cannot be consumed by bacterivores in these pores) or due to water or oxygen availability. Here, the role of soil ecosystem engineers would be crucial. In reality, the structural diversity of a soil is extremely important. A precise model would need to include a full 3D description of the rhizosphere which is beyond the scope of an ecosystem model at the scale we envisage. However, some aspects can be included by simulating root exudates as 100% accessible.

Concerning nutrients, the described model concept is limited to the nutrients available from SOM decay and ignores mineral weathering. Improved understanding of the interactions between the different soil biota and the soil geochemistry could enhance this concept, for example including the weathering effect of mycorrhizal fungi (Andrews et al., 2011), but available studies are as yet limited. For less soluble nutrients such as P depending on the parent material, pH and concentration of base cations, a more chemical approach (including the simulation of pH depending on parent material) might be necessary but hard to parameterize at an ecosystem scale, although Yang et al. (2019) showed this can give good results at a regional scale. In many cases an empirical approach as used in Bortier et al. (2018) could be added, for example for podzol soils where nutrient availability is low.

For the faunal food web, we have chosen to represent functional groups, instead of species. For the parameterization of these groups, average values of the main species can be used, as described in the sections above.

We describe a single layer here, but it is the goal to simulate the distinct horizons of a soil, since using average values when the soil horizons are strongly differentiated induces large errors. For hydrological simulations distinct soil layers need to be distinguished even if their composition is similar.

## Conclusions

Recent technological advances such as high-throughput DNA sequencing and stable isotopes analyses have greatly increased our knowledge and understanding of the key soil processes and how they interlink. Yet, the key interactions between major actors in the soil are often ignored in widely used soil models, and are only represented in complex models, focusing only on specific processes but not on ecosystem functioning.

Our model concept KEYLINK is a novel and simple yet integrative representation of the latest insights from different ‘schools’ of soil description and analyses. By including and linking the major faunal groups, the description of the soil pore space and the active decomposition of SOM, a dynamic link between management, climate and soil functioning is attainable. More insight into the interaction between the different soil biota, soil chemistry and soil structure is required to improve and validate this concept.

The strength of our concept goes beyond getting a more reliable prediction of soil processes. It is clear that, due to the limited available data for many sites, in many cases a very simplistic representation of the soil can, with site-specific parameterization, yield a reasonable fit to measured data. Indeed, given enough parameters and pools, and limited validation data, almost any model can “fit”. However, existing models, in which the growth of plants is limited by soil nutrient and water content only, create the false impression that adding nutrients and water is enough to have a well-functioning ecosystem. This is in contrast to all recent findings concerning the importance of a well-functioning soil ecosystem including a diverse soil fauna that efficiently buffers the nutrient and water availability. Therefore, we believe that our model concept stimulates viewing the soil as a complex living system that needs to be protected in its diversity so it can fulfil all ecosystem functions.

## Additional Information and Declarations

### Data Availability

The following information was supplied regarding data availability: This is a model concept presented in a literature review, there is no code and no data.
